# High-affinity autoreactive plasma cells disseminate through multiple organs in patients with immune thrombocytopenic purpura

**DOI:** 10.1172/JCI153580

**Published:** 2022-06-15

**Authors:** Pablo Canales-Herrerias, Etienne Crickx, Matteo Broketa, Aurélien Sokal, Guilhem Chenon, Imane Azzaoui, Alexis Vandenberghe, Angga Perima, Bruno Iannascoli, Odile Richard-Le Goff, Carlos Castrillon, Guillaume Mottet, Delphine Sterlin, Ailsa Robbins, Marc Michel, Patrick England, Gael A. Millot, Klaus Eyer, Jean Baudry, Matthieu Mahevas, Pierre Bruhns

**Affiliations:** 1 Institut Pasteur, Université Paris Cité, INSERM UMR1222, Unit of Antibodies in Therapy and Pathology, Paris, France.; 2 Laboratoire Colloïdes et Matériaux Divisés (LCMD), ESPCI Paris, PSL Research University, CNRS UMR8231 Chimie Biologie Innovation, Paris, France.; 3 École Doctorale Frontières du Vivant (FdV), Centre de Recherches Interdisciplinaires, Paris, France.; 4 Institut Necker-Enfants Malades, INSERM U1151/CNRS UMS8253, Université Paris Descartes, Sorbonne Paris Cité, Paris, France.; 5 Service de Médecine Interne, Centre national de référence des cytopénies auto-immunes de l’adulte, Hôpital Henri Mondor, Assistance Publique Hôpitaux de Paris (AP-HP), Université Paris Est Créteil, Créteil, France.; 6 Diaccurate SA, Paris, France.; 7 Sorbonne University, ED394, Paris, France.; 8 INSERM U955, Université Paris Est Créteil (UPEC), Créteil, France.; 9 Department of Internal Medicine, Infectious Diseases, and Clinical Immunology, Robert Debré Hospital, Reims University Hospitals, Reims, France.; 10 Centre Hospitalier Universitaire Henri-Mondor, Université Paris Est Créteil, Créteil, France.; 11 Paris Est Créteil University UPEC, Assistance Publique-Hôpitaux de Paris (AP-HP), Henri Mondor Hospital, Fédération Hospitalo-Universitaire TRUE InnovaTive theRapy for immUne disordErs, Créteil, France.; 12 Plateforme de Biophysique Moléculaire, Institut Pasteur, CNRS UMR3528, Paris, France.; 13 Hub Bioinformatique et Biostatistique – DBC, Institut Pasteur, Paris, France.; 14 Laboratory for Functional Immune Repertoire Analysis, Institute of Pharmaceutical Sciences, ETH Zurich, Zurich, Switzerland.

**Keywords:** Autoimmunity, Therapeutics, Adaptive immunity, Autoimmune diseases, Platelets

## Abstract

The major therapeutic goal for immune thrombocytopenic purpura (ITP) is to restore normal platelet counts using drugs to promote platelet production or by interfering with mechanisms responsible for platelet destruction. Eighty percent of patients with ITP possess anti–integrin **α**IIb**β**3 IgG autoantibodies that cause platelet opsonization and phagocytosis. The spleen is considered the primary site of autoantibody production by autoreactive B cells and platelet destruction. The immediate failure in approximately 50% of patients to recover a normal platelet count after anti-CD20 rituximab-mediated B cell depletion and splenectomy suggests that autoreactive, rituximab-resistant, IgG-secreting B cells (IgG-SCs) reside in other anatomical compartments. We analyzed more than 3,300 single IgG-SCs from spleen, bone marrow, and/or blood of 27 patients with ITP, revealing high interindividual variability in affinity for **α**IIb**β**3, with variations over 3 logs. IgG-SC dissemination and range of affinities were, however, similar for each patient. Longitudinal analysis of autoreactive IgG-SCs upon treatment with the anti-CD38 mAb daratumumab demonstrated variable outcomes, from complete remission to failure with persistence of high-affinity anti–**α**IIb**β**3 IgG-SCs in the bone marrow. This study demonstrates the existence and dissemination of high-affinity autoreactive plasma cells in multiple anatomical compartments of patients with ITP that may cause the failure of current therapies.

## Introduction

Immune thrombocytopenic purpura (ITP) is an immune disorder characterized by a strong autoimmune response against platelet autoantigens that causes platelet destruction (1). Clinical manifestations often present as mild bleeding on the skin or mucosal surfaces, but can also include life-threatening internal bleeding episodes in more severe cases (2, 3). The hallmark of ITP is the presence of anti-platelet antibodies, which contributes to the accelerated destruction of platelets by mechanisms that include FcR-mediated phagocytosis by macrophages, and inhibition of new platelet generation by megakaryocytes. Autoantibodies against platelets are predominantly of the IgG isotype (4), with IgG1 being the most prevalent subclass (5). Although several platelet surface proteins are known to be targeted by autoantibodies, namely the CD42 complex (GPIb/IX/V), integrin α2β1 (GPIa/IIa), and GPVI (6), the integrin αIIbβ3 complex (GPIIb/IIIa) has long been known as the dominant autoantigen in ITP, with anti–integrin αIIbβ3 antibodies found in 60% to 90% of patients (6–8).

The spleen plays a major role in the pathophysiology of ITP (9), and splenectomy remains the most effective and curative therapy (10). Indeed, autoreactive anti-αIIbβ3–secreting cells arise from germinal center reactions located in the spleen of patients with ITP. Germinal center expansion results from T follicular helper cell activation (11) and reduced Treg cell numbers (12). Pathogenic anti-αIIbβ3 antibody–secreting cells can be detected by ELISpot in the spleen and blood of patients with ITP (9, 13, 14). In this prototypic antibody-mediated autoimmune disease, B cell depletion using rituximab (anti-CD20 mAb) is widely used, with up to 60% of patients having a short-term complete response (15), 40% achieving durable responses (6–12 months), and only 20% to 30% achieving a long-term response (5 years; refs. 16, 17). Paradoxically, B cell depletion therapy stimulates an adaptation of splenic short-lived plasma cells that leads to their reprogramming into long-lived spleen cells in patients with ITP, explaining in part primary failure of rituximab (13). Furthermore, relapse of ITP after B cell depletion therapy with rituximab usually occurs during B cell lymphopoiesis (more than 6 months after treatment), corresponding to the reinitiation of an autoimmune B cell response. We recently demonstrated that rituximab-resistant memory B cells directly contributed to ITP relapses by enabling autoreactive germinal centers along with the recruitment of naive B cells and leading to anti-αIIbβ3 secretion by newly formed antibody-secreting cells (ASCs) (18).

In a T cell–dependent response such as ITP, ASCs arise from germinal centers or memory B cells and egress the spleen as short-lived ASCs to circulate in the blood, with some maturing into long-lived plasma cells in the bone marrow. The longevity of ASCs is not primarily cell intrinsic but largely depends on signals provided by their microenvironment (19–21). Despite their fundamental role, a major impediment to studying heterogeneity of autoimmune ASCs is the difficulty in assessing the specificity of such cells that express no or relatively few immunoglobulins at their surface. In order to decipher the exact contribution of different subsets of anti-αIIbβ3 ASCs to the pathogeny in different organs and in different clinical situations, we performed herein a high-throughput phenotypic analysis of these autoreactive human IgG-secreting cells (IgG-SCs) at single-cell level, comparing patients with ITP and healthy donors. We describe a 3-log repertoire of affinities of anti-αIIbβ3 IgG autoantibodies secreted from freshly isolated spleen, bone marrow, and blood. The proportion of autoreactive cells among IgG-SCs was highly correlated between the spleen and the 2 other compartments, and very-high-affinity anti-αIIbβ3 IgG-SCs were detected in all 3 compartments. A longitudinal analysis of autoreactive IgG-SCs in 3 patients refractory to approved treatments who were administered the anti-CD38 mAb daratumumab demonstrated interindividual variability in the targeting of high-affinity anti-αIIbβ3 IgG-SCs. These studies identify the large repertoire of anti-αIIbβ3 affinities in patients with ITP and demonstrate the existence and dissemination of high-affinity autoreactive ASCs to the bone marrow of patients with ITP that may be the underlying cause of failure of current therapies.

## Results

### Single-cell bioassay allows phenotypic characterization of autoreactive ASCs.

**Figure 1:**
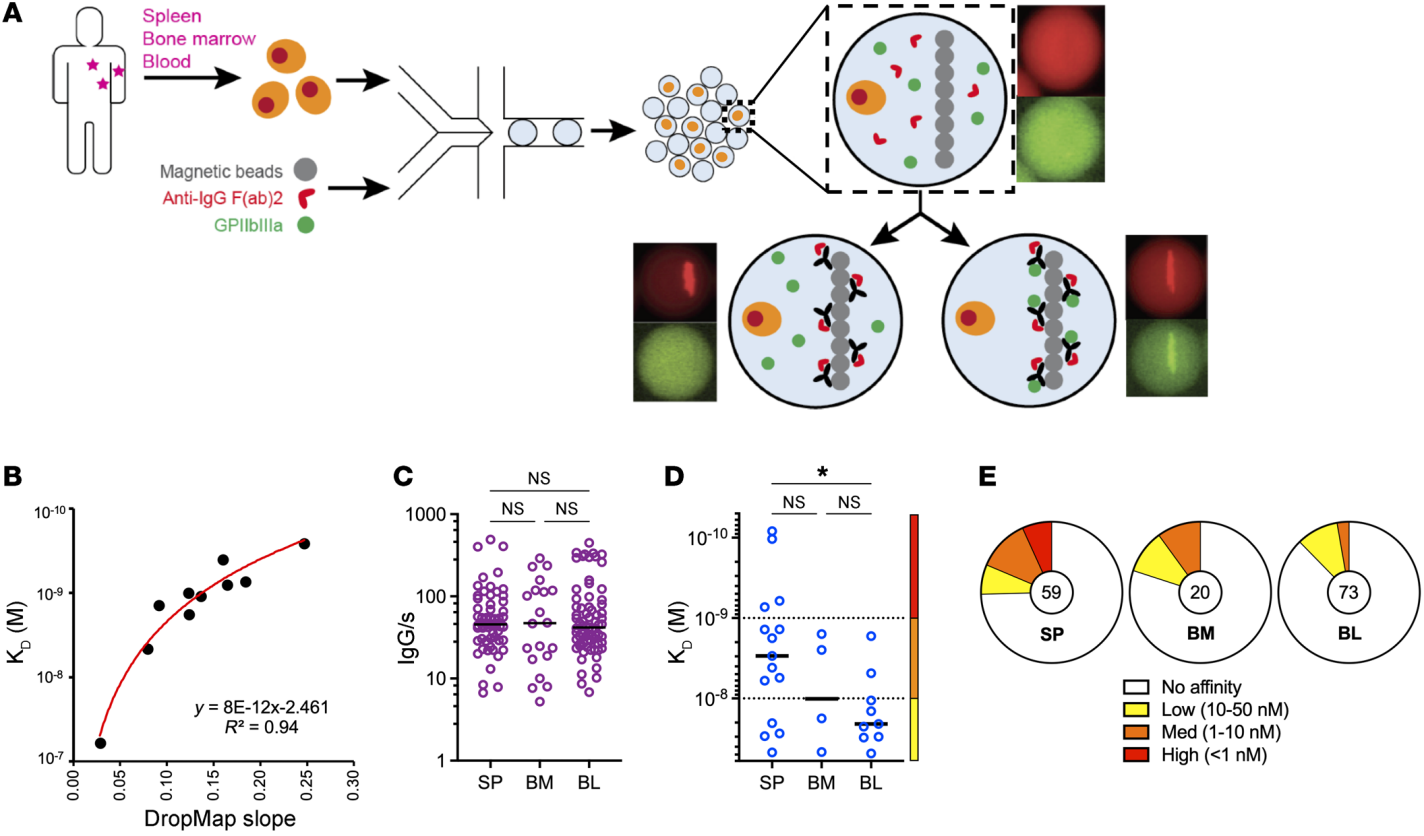
Single-cell analyses reveal αIIbβ3 affinity and secretion rate from IgG-SCs. (**A**) Schematic of the DropMap pipeline. Single mononuclear cells are encapsulated in droplets, together with magnetic beads coated with anti–κ light chain nanobody (VHH) and fluorescently labeled anti-IgG F(ab′)_2_–Alexa Fluor 647 (red) and αIIbβ3–Alexa Fluor 488 (green). Droplets are immobilized in the chip, exposed to a magnetic field to induce beads to form a beadline, and imaged over time. (**B**) αIIbβ3 affinity reference curve generated using anti-αIIbβ3 mAbs with known *K_D_* and values obtained from DropMap experiments using these mAbs. (**C**–**E**) DropMap analysis of spleen (SP), bone marrow (BM), and blood (BL) samples from patient F at the time of splenectomy. (**C**) IgG secretion rate. (**D**) Affinity for αIIbβ3 (*K_D_*) classified into high (red), medium (orange), and low (yellow) affinity, with dotted lines separating these categories. (**E**) Distribution of IgG-SCs into low (yellow), medium (orange), and high (red) affinity binders to αIIbβ3 or nonbinders (white), with total IgG-SC numbers indicated. (**C** and **D**) Single-cell values and medians are plotted. *P < 0.05 using Welch’s *t* test and multiple-testing *P*-value adjustment. NS, not significant. See Supplemental Table 2 for further details.

In order to directly characterize the secretion rate, specificity, and affinity for αIIbβ3 of IgG secreted by autoreactive plasma cells and plasmablasts, collectively termed ASCs hereafter, without the need to sort, clone, or re-express antibodies, we adapted a single-cell bioassay in microfluidic droplets (termed “DropMap”) that we have described previously (22, 23). In this assay, mononuclear cells from the spleen, bone marrow, or blood of patients and fluorescent bioassay reagents are coencapsulated in droplets, immobilized within an observation chamber, and imaged over 1 hour by time-lapse fluorescence imaging. Magnetic beads in each droplet form a line (beadline) under a magnetic field to serve as a physical surface for a double-fluorescent sandwich ELISA, revealing IgG secretion from the cell and specificity of that IgG for αIIbβ3 (Figure 1A). The time-resolved fluorescence signals allow for estimation of IgG secretion rates and affinity for αIIbβ3 of the secreted IgG by using calibration curves generated with monoclonal anti-αIIbβ3 IgG of known affinity (*K_D_*). The anti-αIIbβ3 reference curve, generated using 9 anti-αIIbβ3 IgG mAbs of various affinities, allows for measurements over 2 logs of affinities, i.e., 2.5 × 10^–10^ ≤ *K_D_* ≤ 5 × 10^–8^ M (Figure 1B). Therefore herein, IgG interacting with αIIbβ3 at a calculated *K_D_* below 5 × 10^–8^ M was considered binding, i.e., anti-αIIbβ3 IgG antibodies, and the cells secreting such IgG were termed “αIIbβ3-reactive ASCs.”

As an example, samples from the spleen, bone marrow, and blood were obtained at the time of splenectomy from 1 patient (patient “F”; 73-year-old male) who was splenectomized 8 months after the last rituximab infusion due to treatment failure (Figure 1, C–E). All data from these samples were acquired in duplicate on the same day with high reproducibility in total and autoreactive IgG-SC detection per replicate (Supplemental Figure 1; supplemental material available online with this article; https://doi.org/10.1172/JCI153580DS1). For each organ we analyzed 5,000–9,000 single cells in total, and found IgG-SCs represented 0.55%, 0.41%, and 1.26% of the mononuclear cell pool in the spleen, bone marrow, and blood, respectively. IgG-SCs from the 3 organs displayed a similar range (5–500 molecules per second [IgG/s]) and median values (approximately 50 IgG/s) of IgG secretion (Figure 1C). Within the IgG-SC pool of all 3 organs, a fraction secreted anti-αIIbβ3 IgG antibodies with *K_D_* values in the 5 × 10^–8^ M to 1 × 10^–10^ M range that we categorized into low-affinity (50 nM ≤ *K_D_* ≤ 10 nM), medium-affinity (10 nM < *K_D_* ≤ 1 nM), and high-affinity (*K_D_* < 1 nM) αIIbβ3-reactive IgG-SCs to facilitate subsequent analyses (Figure 1D). In this patient, the spleen contained IgG-SCs with significantly higher affinities for αIIbβ3 as compared with the blood, and the proportion of αIIbβ3-reactive cells among IgG-secreting cells was 25% in the spleen, 20% in the bone marrow, and 12% in the blood, with high-affinity antibodies detected only in the spleen (Figure 1E).

### High interindividual variability of autoreactive ASC presence and affinity in spleen, bone marrow, and blood of patients with ITP.

Our cohort included 25 patients diagnosed with chronic or acute ITP, with a median age of 51 years (ranging 21–79 years), to investigate the anatomical distribution, affinity, and secretion rate of anti-αIIbβ3 IgG-SCs. Clinical characteristics are presented in Table 1. Eight patients (8 of 18) achieved complete response after splenectomy, with a follow-up of 14 to 24 months, while 10 patients (10 of 18) had no significant increase in platelet counts after splenectomy. A bone marrow aspirate was also performed in 7 of 18 patients in addition to the programmed splenectomy. We also analyzed bone marrow and blood from 9 patients with ITP that were not splenectomized (Table 1), and 21 healthy donors (no immune disease) as controls for spleen, bone marrow, or blood samples. For every sample, we analyzed an average of 50,000 to 100,000 droplets in total, representing an average of 10,000 to 27,000 single mononuclear cells containing 0.01%–5% IgG-SCs (Supplemental Figure 2). Compared with the detection of anti-αIIbβ3 IgG-SCs by ELISpot, DropMap was far more sensitive (Supplemental Figure 3A).

**Figure 2:**
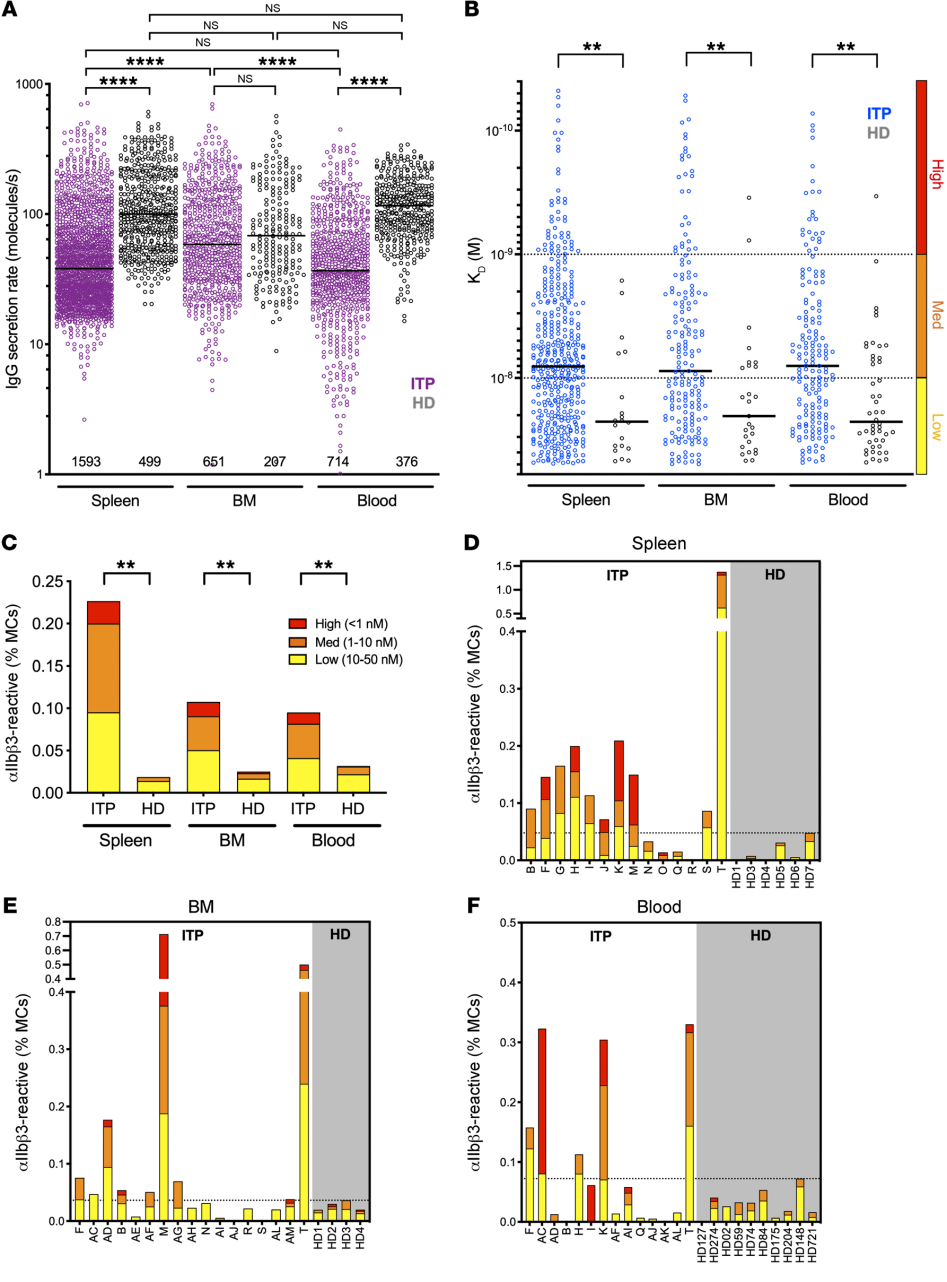
Autoreactive ASCs with high and low affinity are present in the spleen, blood, and bone marrow (BM) of patients with ITP. (**A**) IgG secretion rate and (**B**) affinity for αIIbβ3 of single ASCs from pooled data of patients with ITP and healthy donors (HD) for spleen (ITP, *n =* 14; HD, *n =* 6), BM (ITP, *n =* 17; HD, *n =* 4), and blood (ITP, *n =* 14; HD, *n =* 10). Single-cell values and medians are plotted. Total numbers of IgG-SCs analyzed per compartment are indicated in **A**. In **B**, affinities are classified into high (red), medium (orange), and low (yellow) affinity, with dotted lines separating these categories. (**C**) Frequency of αIIbβ3-reactive IgG-SCs among mononuclear cells (MCs) classified into high, medium, and low affinity from patients (ITP) and HD according to **B**. (**D**–**F**) Frequency of αIIbβ3-reactive IgG-SCs among MCs classified into high, medium, and low affinity represented for individual patients (ITP) and HD for (**D**) spleen, (**E**) BM, and (**F**) blood. (**D**–**F**) The dotted line marks the highest frequency found in an HD. **P < 0.01; ****P < 0.0001 using post hoc contrast analysis after linear (**A** and **B**) or logistic (**C**) modeling and multiple-testing *P*-value adjustment. NS, not significant. See Supplemental Table 2 for further details.

We first analyzed the global IgG-SC response per organ for all patients and healthy donors and found that among mononuclear cells, 0.01% to 5% had detectable levels of IgG secretion, with a wide range of IgG secretion rates (1–713 IgG/s) (Figure 2A and Supplemental Figure 2C). In healthy donors, IgG secretion rates were not significantly different between the spleen, blood, and bone marrow. However, in patients with ITP the IgG secretion rates in the bone marrow were significantly higher than in spleen and blood. Unexpectedly, splenic IgG-SCs showed significantly (2.5-fold) lower secretion rates in patients with ITP compared with healthy donors, with median values of 46 IgG/s and 116 IgG/s, respectively. Similar findings were observed for IgG-SCs from peripheral blood, with a 2-fold lower secretion rate in patients with ITP (median 37 IgG/s) compared with healthy donors (74 IgG/s). However, IgG-SCs in the bone marrow had similar secretion rates between patients with ITP and healthy donors (median 60 and 59 IgG/s, respectively) (Figure 2A). In all 3 compartments, the top approximately 20% highest IgG producers were responsible for approximately 50% of the total amount of secreted IgG (Supplemental Figure 3, B and C).

Similar to the first patient we analyzed as a proof of concept (Figure 1, C–E), we identified IgG-SCs with affinity in the 5 × 10^–8^ M to 5 × 10^–11^ M range for αIIbβ3 in pooled data from all 3 anatomical sites of patients with ITP, but also in significantly fewer numbers in pooled data from healthy donors in the 5 × 10^–8^ M to 5 × 10^–9^ M range (Figure 2B). Only patients with ITP had IgG-SCs with very high estimated affinity (*K_D_* below 10^–10^ M), mathematically extrapolated from values outside the boundaries of the reference curve presented in Figure 1B. A weak (low *R* value) but significant positive correlation was found between *K_D_* and secretion rate using the pooled data, suggesting that high-affinity autoreactive ASCs tend to secrete less than low-affinity autoreactive ASCs (Supplemental Figure 3, D–F). No correlation, however, was found between time elapsed since the end of rituximab treatment and either secretion rate or affinity in any compartment analyzed (Supplemental Figure 4). Only a fraction (8%–13%) of IgG-SCs showed cross-binding to αIIbβ3 and antigens used for polyreactivity testing (keyhole limpet hemocyanin [KLH] and insulin; ref. 24) with poor affinity (10–50 nM), as expected from IgG-secreting plasma cells (25), and increased affinity for αIIbβ3 did not increase affinity for these other antigens (Supplemental Figure 5). These results demonstrate that approximately 90% of the IgG-SCs with reactivity for αIIbβ3 analyzed herein are not polyreactive. The overall median affinity of anti-αIIbβ3 IgG-SCs from pooled patients with ITP was identical (~8 nM) between the 3 anatomical compartments (Figure 2B). The same result was found for anti-αIIbβ3 IgG-SCs of pooled healthy donors, with a significantly lower median affinity (~23 nM) (Figure 2B). The proportion of autoreactive IgG-SCs among mononuclear cells was 10-fold, 5-fold, and 3-fold higher in patients with ITP than healthy donors in the spleen, bone marrow, and blood, respectively (Figure 2C). Healthy donors harbored 65% to 75% low-affinity and relatively few high-affinity IgG-SCs, whereas patients with ITP harbored 37% to 46% medium affinity and 11% to 16% high-affinity IgG-SCs in the 3 anatomical compartments analyzed. Compared with healthy donors, patients with ITP secrete IgG at a lower rate but with a higher proportion of medium- and high-affinity anti-αIIbβ3 specificities in spleen, bone marrow, and blood. These results suggest a role for high-affinity autoantibodies secreted from different anatomical locations in the pathogenesis of ITP.

With the majority of the patients in our cohort being refractory to various therapies, including B cell depletion and splenectomy, we wondered whether patients achieving complete response to rituximab therapy would demonstrate a reduction/disappearance of the high-affinity anti-αIIbβ3 IgG-SC population after treatment. We retrospectively analyzed frozen PBMC samples of 4 responder ITP patients collected 1 week before the first and 3 months after the last rituximab infusion. Seventy-five percent (3 of 4) patients displayed 3 times or greater less total IgG-SCs after rituximab treatment, with nevertheless a nonsignificant trend for the group (Supplemental Figure 6A). IgG secretion rates were, however, significantly higher after treatment (Supplemental Figure 6B), reminiscent of the higher secretion rate of blood IgG-SCs in healthy donors compared with patients with ITP (Figure 2A). All 4 responder patients had very low initial frequencies of αIIbβ3-specific IgG-SCs in circulation (mean 0.016% among PBMCs), with approximately 10-fold reduction in 2 patients and no major variation in the 2 other patients (Supplemental Figure 6C). Nevertheless, high-affinity autoreactive IgG-SCs disappeared after treatment in these patients in remission (Supplemental Figure 6D), supporting our hypothesis on the role for high-affinity ASCs in the pathogenesis of ITP. We then grouped the patients from our main cohort by rituximab responsiveness and compared them in terms of αIIbβ3 reactivity. Even though the number of responding patients in our cohort was very limited, we observed a tendency toward lower affinity for αIIbβ3 in rituximab-responding patients as compared with rituximab-failure patients (Supplemental Figure 6E), whereas no difference was observed in the frequency of autoreactive ASCs (Supplemental Figure 6F).

We found varying proportions of autoreactive IgG-SCs among mononuclear cells between patients with ITP, and within anatomical compartments of a given patient (Figure 2, D–F). This finding may rely on the heterogeneity of therapeutic regimens and intrinsic cell heterogeneity of each anatomical compartment. Nevertheless, 71% (10 of 14) of spleen samples, but only 41% (7 of 17) of bone marrow and 36% (5 of 14) of blood samples harbored more autoreactive IgG-SCs in patients with ITP compared with the highest value found in healthy control samples. Fifty percent (7 of 14) of the patients with ITP harbored high-affinity anti-αIIbβ3 IgG-SCs in the spleen, compared with 29% (5 of 17) and 36% (5 of 14) in the bone marrow and blood, respectively. No such cell could be detected in spleens from healthy donors, and only once in blood and twice in bone marrow at very low numbers. Thus, most patients with ITP displayed a robust anti-αIIbβ3 response in the spleen that underlies the central role of this organ in ITP.

### Comparable autoantibody responses in paired organs.

**Figure 3:**
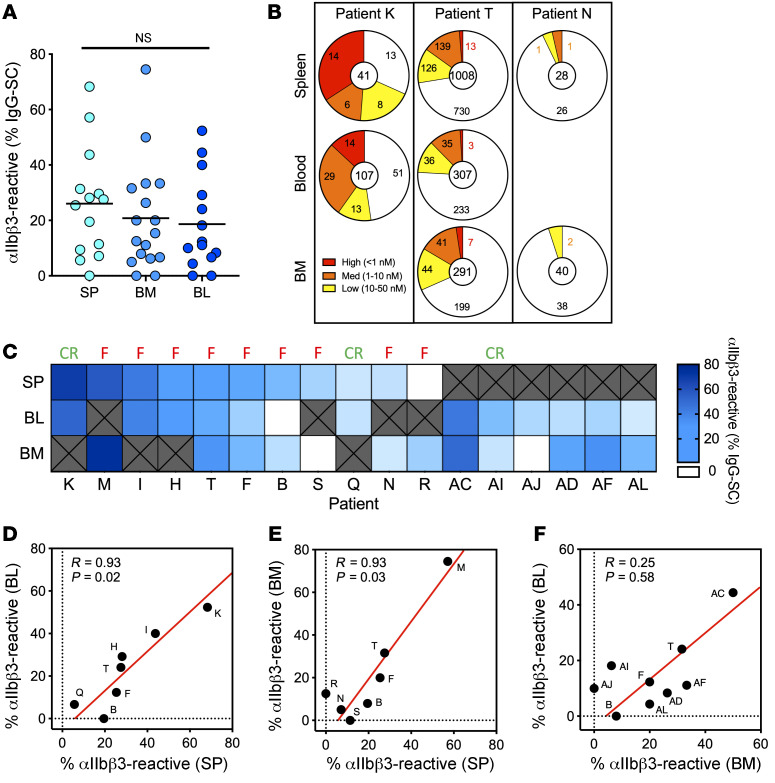
Paired organs demonstrate a comparable autoantibody response. (**A**) Frequency of αIIbβ3-reactive IgG-SCs among total IgG-SCs in the spleen (SP), bone marrow (BM), and blood (BL) from individual patients with ITP. Each dot represents an individual. Values are pooled data from 2 replicates. NS, not significant using Welch’s *t* test and multiple-testing *P*-value adjustment. See Supplemental Table 2 for further details. (**B**) Distribution of IgG-SCs into low (yellow), medium (orange), and high (red) affinity binders to αIIbβ3 or nonbinders (white), with total IgG-SC numbers indicated, for paired samples of patients K, T, and N. (**C**) Heatmap of the frequency of αIIbβ3-reactive cells among IgG-SCs per patient, ordered from most frequent to less frequent in spleen for patients with a spleen sample, and ordered from most frequent to less frequent in blood for patients without a spleen sample. White boxes indicate undetectable reactivity for αIIbβ3, and “X” indicate absence of samples. Result of splenectomy is indicated: complete remission (CR) or failure (F). (**D**–**F**) Pearson’s correlation analysis of data from **C**; scatterplots compare the frequency of αIIbβ3-reactive cells among paired organs. *R* and adjusted *P* values are indicated. Red line indicates the reduced major axis.

In order to compare the autoantibody response in different immune sites from the same patient with ITP, we obtained paired samples, either taken at the time of splenectomy (spleen + bone marrow and/or blood) or at the time of bone marrow collection (bone marrow + blood). On average, 20% to 25% of IgG-SCs were αIIbβ3 specific in the 3 compartments, with some patients presenting with a robust autoreactive response of up to 75% of autoreactive IgG-SCs among all IgG-SCs (Figure 3A). Distribution of affinities for IIbβ3 was similar within compartments of individual patients, as exemplified in Figure 3B (refer to Supplemental Figure 7 for all paired samples). For example, patient K displayed a robust anti-αIIbβ3 response, with greater than 50% autoreactive IgG-SCs distributed among high-, medium-, and low-affinity IgG-SCs in both spleen and blood; patient T displayed large numbers of IgG-SCs, with approximately 25% autoreactive IgG-SCs largely predominated by low- and medium-affinity IgG-SCs in all 3 compartments; and patient N displayed low numbers of IgG-SCs, with approximately 5% autoreactive IgG-SCs (Figure 3B). Generally, in the cohort of paired samples, patients harboring a large proportion of αIIbβ3-reactive IgG-SCs among all IgG-SCs in one compartment also did so in the other one or two compartments (Figure 3C). For the 7 patients for whom splenectomy failed to induce clinical remission and a bone marrow sample was available (patients M, T, F, B, S, N, and R), anti-αIIbβ3 IgG-SCs were present in the bone marrow the day of splenectomy, except for patient S. This suggests that the autoreactive bone marrow ASC population is responsible for the sustained disease observed after splenectomy in these patients.

Proportions of αIIbβ3-reactive IgG-SCs correlated well between spleen and blood (*R* > 0.9; *P =* 0.02) and spleen and bone marrow (*R* > 0.9; *P =* 0.03), but did not correlate between bone marrow and blood (*R* = 0.25; *P =* 0.58) (Figure 3, D–F). Despite the heterogeneity of clinical situations and treatment history among patients in this cohort, the spleen appears to determine the extent of the autoreactive response, likely by providing the other compartments with autoreactive IgG-SCs. Our results suggest that the autoreactive-IgG response in patients with ITP disseminates through multiple organs, resulting in IgG-SC populations with similar ranges of anti-αIIbβ3 affinity and proportion among IgG-SCs.

### Kinetic follow-up of anti-CD38 therapy with daratumumab in patients with ITP.

**Figure 4:**
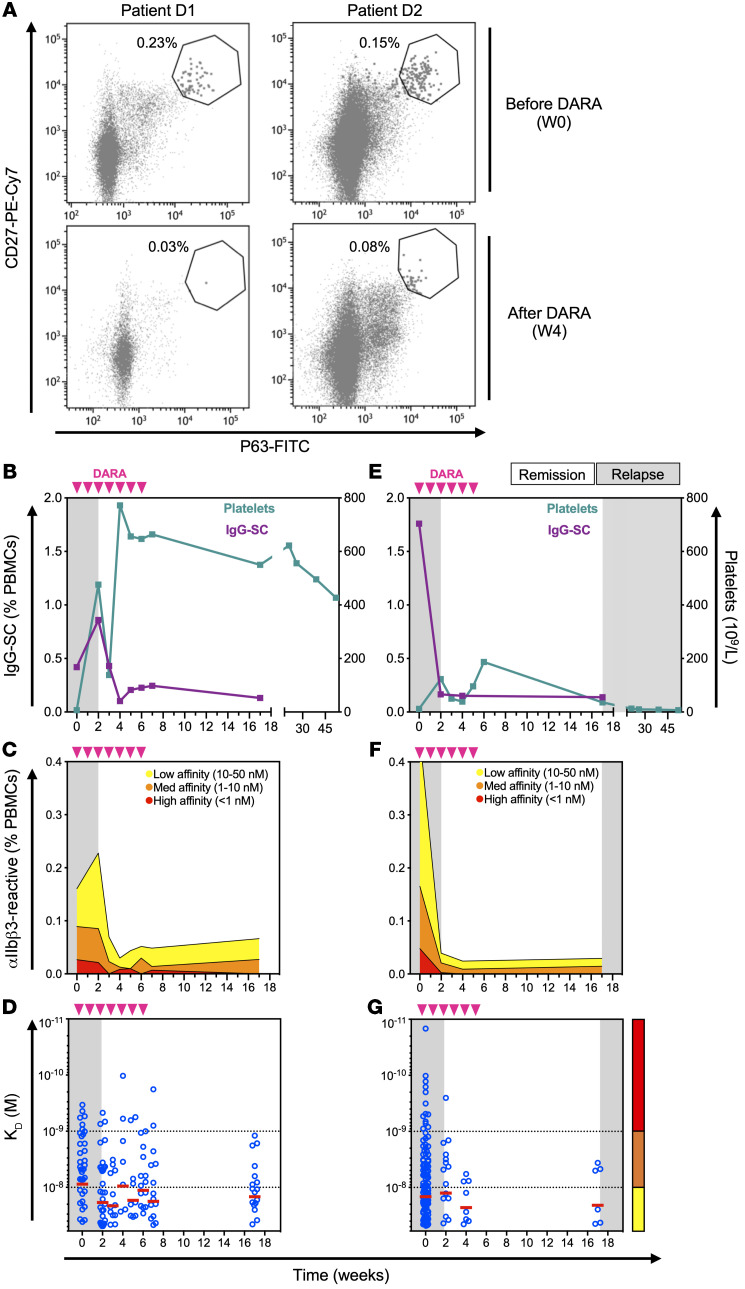
Anti-CD38 therapy depletes pathogenic ASCs in patients with ITP. (**A**) Flow cytometric identification of circulating CD27^+^P63^+^ plasmablasts/plasma cells from patients D1 and D2 before (top panel) and 4 weeks after (bottom panel) the start of daratumumab (DARA) treatment. (**B**–**G**) Kinetic follow-up of patient D1 (**B**–**D**) and patient D2 (**E**–**G**) blood samples for (**B** and **E**) frequency of IgG-SCs among PBMCs and platelet levels. (**C** and **F**) Frequency of αIIbβ3-reactive IgG-SCs among PBMCs classified into high (red), medium (orange), and low (yellow) affinity. (**D** and **G**) Affinity for αIIbβ3 of single IgG-SCs. Displayed on the background is time spent in clinical remission (white) or relapse (gray). Daratumumab infusions are indicated by pink arrows. Single-cell affinity values and medians are plotted in **D** and **G**.

The autoreactive IgG-SCs in patients with chronic ITP that are refractory to conventional therapies (rituximab and/or splenectomy) can be theoretically eliminated using the anti-CD38 mAb daratumumab that was developed to target malignant plasma cells (26). We therefore analyzed 3 patients who received off-label (compassionate use) daratumumab for severe chronic refractory ITP. After 3 infusions, daratumumab led to an 89% reduction in circulating CD27^+^P63^+^ plasmablasts and plasma cells (27), identified by flow cytometry (Figure 4A and Supplemental Figure 8).

Patient D1 (34-year-old male) had been splenectomized 9 years prior to daratumumab therapy, but continued to experience skin and/or mucosal bleedings because of low platelet counts (<30 × 10^9^/L) despite receiving several immunosuppressant drugs (including rituximab, mycophenolate mofetil, and cyclosporin) and thrombopoietin receptor agonist (TPO-RA). He received 7 daratumumab infusions at 16 mg/kg per week without other treatment except oral dexamethasone (20 mg) before each infusion and achieved a complete response lasting now over 1 year (defined by platelet count >100 × 10^9^/L) (Figure 4B). After a short increase in total IgG-SC (Figure 4B) and anti-αIIbβ3 IgG-SC (Figure 4C) numbers in blood, these proportions dropped 2- to 3-fold and remained low for at least 10 weeks after the last daratumumab infusion. Remarkably, whereas low-affinity IgG-SCs remained at a third of their initial level, high-affinity IgG-SCs became undetectable a few weeks after the end of the treatment (Figure 4, C and D).

Patient D2 (35-year-old female) had not been splenectomized and received her last rituximab infusion 8 years before receiving weekly daratumumab (without other treatment except 20 mg dexamethasone orally before each infusion) for 6 weeks. This resulted in a transient complete response lasting 17 weeks before relapse. Total IgG-SC (Figure 4E) and anti-αIIbβ3 IgG-SC numbers in blood nevertheless dropped approximately 10-fold following daratumumab infusions, again with the disappearance of high-affinity IgG-SCs (Figure 4, F–G). Overall, both patients responded similarly to daratumumab therapy in terms of depletion of IgG-SCs, both total and αIIbβ3 specific, and elimination of high-affinity anti-αIIbβ3 IgG-SCs from circulation.

These results emphasize that daratumumab therapy targets ASCs in patients with ITP and suggest that the depletion of these cells, which include the autoreactive population, could be related to clinical improvement.

### Spleen-independent reappearance of high-affinity anti-αIIbβ3 ASCs after daratumumab.

**Figure 5:**
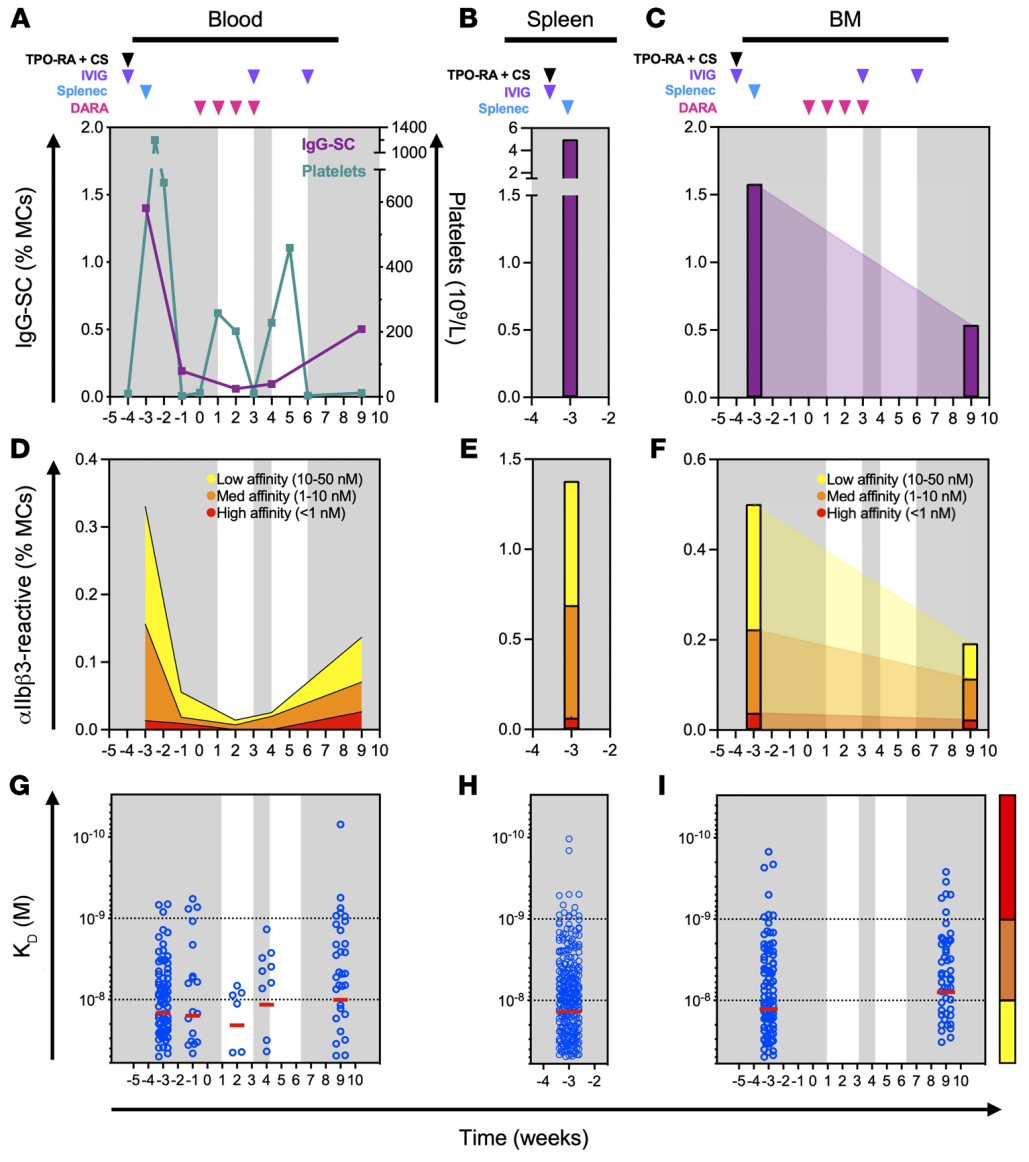
Failure of anti-CD38 treatment is associated with persistence of pathogenic ASCs. Kinetic follow-up of patient T. Displayed on the background is time spent in clinical remission (white) or relapse (gray). Drug infusions are indicated by arrows: thrombopoietin receptor agonist and cyclosporin (TPO-RA + CS; black), intravenous immunoglobulin (IVIG; purple), and daratumumab (DARA; pink). The time of splenectomy is indicated by a blue arrow. (**A**–**C**) Frequency over time of IgG-SCs among mononuclear cells (MCs) in (**A**) blood with platelet levels indicated, (**B**) spleen, and (**C**) bone marrow (BM). (**D**–**F**) Frequency over time of αIIbβ3-reactive IgG-SCs among MCs in (**D**) blood, (**E**) spleen, and (**F**) BM classified into high, medium, and low affinity. (**G**–**I**) Affinity over time for αIIbβ3 of single IgG-SCs in (**G**) blood, (**H**) spleen, and (**I**) BM. Single-cell affinity values (blue dots) and medians (red lines) are plotted.

Patient T (20-year-old female) had ITP requiring splenectomy 10 months after rituximab, and then daratumumab therapy for refractory disease, allowing a sequential follow-up after these interventions. After splenectomy, IgG-SC and anti-αIIbβ3 IgG-SC numbers in blood dropped 7- and 4-fold, respectively (Figure 5, A and D), supporting the spleen as an important source of IgG-SCs in circulation (21, 28). On the day of splenectomy, high numbers of IgG-SCs were found among mononuclear cells in the blood (1.4%), bone marrow (1.6%), and spleen (5%) (Figure 5, A–C), i.e., 7-fold higher than the ITP spleen with the highest numbers we had analyzed before (Supplemental Figure 2). Among IgG-SCs, 27%, 32%, and 24% were αIIbβ3 specific, including high-affinity IgG-SCs, in the spleen, bone marrow, and blood, respectively, demonstrating a relatively homogeneous distribution of autoreactive IgG-SCs across all 3 compartments (Figure 5, D–I). Daratumumab treatment started 3 weeks after splenectomy induced a transient increase in platelet counts (Figure 5A), associated with a decrease in total IgG-SC and anti-αIIbβ3 IgG-SC numbers (Figure 5D), and led to a disappearance of high-affinity IgG-SCs (Figure 5G). Daratumumab was discontinued after 4 infusions because of relapse and an increase in total IgG-SCs, anti-αIIbβ3 IgG-SCs, and high-affinity IgG-SCs in the blood was observed after 5 weeks (Figure 5, A, D, and G). This reappearance of autoreactive, high-affinity IgG-SCs in circulation occurred after splenectomy, suggesting autoreactive B cell reservoirs were present in other anatomical compartments or re-emergence of autoreactivity from naive B cells in secondary lymphoid organs (e.g., lymph nodes) (18). Remarkably, whereas bone marrow IgG-SCs (Figure 5C) and anti-αIIbβ3 IgG-SCs (Figure 5F) were reduced 2.5- to 3-fold compared with their content before splenectomy and daratumumab treatment, high-affinity IgG-SCs could be readily detected in the bone marrow 5 weeks later with a similar distribution (Figure 5I). These bone marrow–resistant autoreactive high-affinity IgG-SCs identified after multiple therapies could correspond to daratumumab-resistant ASCs, and/or to newly immigrating ASCs generated in another compartment that remains to be identified.

## Discussion

This study represents the first characterization to our knowledge of ASCs in humans in terms of their anatomical distribution, antibody secretion, and affinity for their target autoantigen, including both patients with autoimmune disease and healthy donors. It demonstrates the dissemination of autoreactive IgG-SCs among the spleen, blood, and bone marrow from patients with ITP. Rates of IgG secretion per cell were very diverse, with a tendency for secretion to decrease with decreasing affinity values for αIIbβ3, but globally similar between IgG-SCs from the spleen and blood and dissimilar from IgG-SCs from the bone marrow. Whereas affinities for the platelet autoantigen αIIbβ3 varied over 3 logs, the median affinity and range of affinities were strikingly similar between the 3 compartments, suggesting a common origin. In addition, the proportion of autoreactive IgG-SCs closely correlated between spleen and blood, and spleen and bone marrow, supportive of the spleen being the source of autoreactive IgG-SCs and the dominant tissue in this disease (9). The persistence of autoreactive ASCs in the bone marrow after failure of anti-CD20 B cell depletion, splenectomy, and anti-CD38 therapy represents a new layer of complexity and target for ITP therapy.

Chronic ITP is the most common hematologic indication for splenectomy (29), making this major secondary lymphoid organ accessible for research. αIIbβ3-reactive memory B cells (13, 18) and IgG-SCs (plasma cells) were identified in the spleen of patients with ITP, as well as increased αIIbβ3-reactive effector T cell (9) and reduced Treg (12) numbers compared with healthy controls. The stage of B cell development at which tolerance to platelet antigens breaks down remains to be determined. The identification in small numbers of anti-αIIbβ3 IgG-SCs in all healthy donors in this work supports the hypothesis of a low-level autoreactivity against platelets in healthy individuals. This is in agreement with the low frequency (~13%) of self-reactive IgG-secreting plasma cells found in healthy donors after sorting of CD138^+^CD27^+^CD38^+^ cells and in vitro expression of their IgG (25). As the abundance of anti-αIIbβ3 IgG-SCs is low, with poor affinity and IgG secretion rates, the amount and overall affinity of circulating platelet autoantibodies may be insufficient in these healthy donors to have a meaningful impact on platelet numbers, but may represent a biomarker for future evolution into ITP.

In ITP, breakdown of tolerance to platelets leads to the generation of anti-αIIbβ3 ASC clones that are a hallmark of germinal center autoreactive B cell generation (11, 13, 18). We provided here a comprehensive view of affinity-matured autoreactive ASCs in different compartments that possess a broad range of affinities, including high-affinity binders. Autoantibodies with affinities for cytokines as high as 3 × 10^–14^ M have been reported to develop in patients with autoimmune polyendocrine syndrome type 1 (APS-1, also known as autoimmune polyendocrinopathy–candidiasis–ectodermal dystrophy [APECED]) (30). Whether antibody affinities for the extremely abundant antigen αIIbβ3 could reach such values remains speculative and may certainly require an extreme defect in tolerance. Remarkably, anti-αIIbβ3 subnanomolar affinities could be identified from IgG-SCs in spleen, blood, and bone marrow in similar distributions in some individuals. The magnitude of the response in the spleen correlated with that of the blood and bone marrow, with a strong decrease in circulating IgG-SCs after splenectomy, highlighting the role of this organ in the generation of IgG-SCs that are released to the bloodstream. Supportive of this notion, a correlation in the number of αIIbβ3-reactive IgG-SCs between spleen and blood from patients with ITP that responded to splenectomy was reported previously using ELISpot (9).

Only 1 case report previously identified anti-αIIbβ3 in the bone marrow of 1 ITP patient using ELISpot (31). Our study identified anti-αIIbβ3 IgG-SCs in the bone marrow in 40% of patients with ITP in similar or higher proportions among mononuclear cells than in the spleen. In a direct comparison, the sensitivity of DropMap was overall higher than ELISpot. In this study, we found the frequency of autoreactive IgG-SCs among mononuclear cells to be around 0.1%, which is 10 times higher than what was previously described using ELISpot (9). This may be due to the inherent higher sensitivity of fluorescent bioassays compared with chromogenic assays, or to the necessity of IgG-SCs to secrete over longer periods of time for ELISpot (hours) than for DropMap (45 minutes). The bone marrow is thought to be the main niche for survival of long-lived ASCs, so the presence of autoreactive IgG-SCs in this compartment could be correlated to long-term autoantibody production and, in turn, with the maintenance of chronic disease (reviewed in ref. 32). Our results constitute one of the first direct findings to our knowledge of autoreactive plasma cell populations in human autoimmunity (33), supported also by similar findings in mouse autoimmunity models of vasculitis (34), lupus (35–38), and encephalomyelitis (39). The establishment of long-lived ASCs in the bone marrow could also have important implications for treatment of ITP and other B cell–dependent autoimmune disorders: first, this population could sustain autoantibody levels following splenectomy, which may be sufficient to affect clinical manifestations; and second, long-lived ASCs in the bone marrow have been observed to be resistant to immunosuppressive agents (36) and to B cell–targeted therapies, such as anti-CD20 or anti-BAFF antibodies (32, 39), which may partly explain therapeutic failures.

In this context, targeting ASCs with anti-CD38 appeared to be a very promising option. This work reports anti-CD38 daratumumab off-label use in 3 patients with refractory chronic ITP, a treatment that has been proposed for autoimmune cytopenia following bone marrow transplantation (40, 41). Similar to our results, daratumumab therapy gave drastically different clinical outcomes, from complete remission to failure of therapy (41). The bone marrow is probably responsible for immediate failures of splenectomy by harboring sufficient autoreactive ASCs for effective platelet destruction. However, targeting autoreactive ASCs may prove difficult, as bone marrow cells have been reported in mouse models to be rather resistant to therapeutic antibody depletion (42, 43). Similarly, we showed that autoreactive ASCs persisted in the bone marrow of a patient with no response to daratumumab, providing a possible explanation for treatment resistance. Another patient achieved complete remission after daratumumab but relapsed several weeks later, suggesting that a lymphoid organ was perhaps able to generate new autoreactive ASCs. The lymphoid organ responsible may have been lymph nodes in splenectomized patient T (Figure 5) and the spleen (and perhaps also lymph nodes) in patient D2 who received daratumumab but had not been splenectomized (Figure 4, E–G). Understanding the reasons for such variability in clinical responses to B cell depletion therapy will require more investigation, particularly as we describe herein a relatively homogeneous dissemination of IgG-SCs among spleen, blood, and bone marrow of patients with ITP.

Our work also has several limitations, mostly inherent to human studies. Patients had varying clinical histories and received treatments that probably affected the ASC pool. However, long-term corticosteroids and/or immunosuppressant drugs are not commonly given for ITP in France, and we took advantage of the various therapeutic sequences, including splenectomy and daratumumab, to study ASC dissemination in humans. Our assay focused on IgG and αIIbβ3, as previous studies showed that anti-αIIbβ3 IgGs were predominant autoantibodies in ITP (4, 5). We cannot, however, exclude the possibility that some patients had a response of another isotype or directed against other, less common, platelet antigens (e.g., CD42 complex [GPIb/IX/V] and integrin α2β1 [GPIa/IIa]; ref. 44). ASCs are also rare among mononuclear cells, and in some patients described here only few αIIbβ3-reactive cells could be identified that limit broad extrapolations on autoreactive ASCs. Our method of analysis proved nevertheless more sensitive than conventional assays (i.e., ELISpot), which allowed us to investigate secretion rate and affinity for αIIbβ3 for more than 3,300 freshly isolated ex vivo IgG-SCs.

This work extends (45–48) our knowledge of autoreactive IgG-SCs in human ITP. We demonstrate the homogeneous dissemination of platelet autoreactive ASCs into multiple anatomical compartments that could explain splenectomy failure in some patients. A wide range of affinities for the major ITP autoantigen αIIbβ3 were identified, with very high affinities reminiscent of affinities found in other systemic autoimmune diseases. Anti-CD38 daratumumab therapy allowed us to demonstrate the crucial contribution of ASCs in ITP, and to point toward a compartment other than the spleen that may serve in some patients as a source to reconstitute autoreactive ASC pools in circulation and in the bone marrow.

## Methods

### ITP patients and controls

All patients included in this study were adults (>18 years old) and were diagnosed with ITP according to international guidelines (10). Patients with underlying immunodeficiencies, hepatitis C virus or human immunodeficiency virus infection, lymphoproliferative disorders, and defined systemic lupus erythematosus (≥4 American Rheumatism Association criteria) were excluded. Patients had acute (<3 months), persistent (3–12 months), or chronic (>12 months) ITP. According to these guidelines, a bone marrow smear biopsy was performed in patients over 60 years old to exclude myelodysplastic syndromes. Complete response to splenectomy was defined by a platelet count over 100 × 10^9^/L and the absence of bleeding, partial response was defined by a platelet count over 30 × 10^9^/L and below 100 × 10^9^/L and at least doubling values from baseline, and failure by a platelet count under 30 × 10^9^/L or use of salvage therapy after 1 month. Relapsing patients were defined as those that initially had a complete response, but then had a drop in the platelet count, below 30 × 10^9^/L, as well as a medical intervention by the treating physician. Healthy controls were individuals with no autoimmune disease or lymphoma. Control spleen samples were obtained from organ donors that died from stroke or head trauma. Control bone marrow aspirate samples were taken from healthy organ transplantation donors and were obtained from the Pitié-Salpêtrière Hospital (AP-HP). Control blood samples were obtained from the French Blood Establishment (EFS).

### Sample processing

Splenic tissue was obtained from splenectomy and maintained at 4°C for transportation to the laboratory. Splenic tissue fragments were homogenized using a dissociator (gentleMACS, Miltenyi Biotec), and cell suspensions were subsequently filtered and diluted in RPMI-1640 (Invitrogen). Ficoll density gradient (Ficoll Paque Plus, GE Healthcare) centrifugation at 300 *g* for 20 minutes with no brake was used to obtain mononuclear cells. Bone marrow cells were obtained from bone marrow aspiration and immediately placed in sodium heparin tubes (BD) for transportation to the lab at room temperature. Bone marrow cells were then dissociated by flushing thoroughly through a syringe needle and diluted in RPMI-1640, before processing by Ficoll density gradient to obtain mononuclear cells. Blood was obtained in sodium heparin tubes and transported at room temperature. After dilution in RPMI-1640, blood was processed by Ficoll density gradient. After isolation, mononuclear cells from all organs were resuspended in RPMI-1640 supplemented with 10% HyClone FBS (Thermo Fisher Scientific) and 1% penicillin/streptomycin (Thermo Fisher Scientific). From this point on, cell suspensions were maintained on ice. All experiments were performed within 12 hours from sample collection, except for PBMCs from 4 patients with nonrefractory ITP that were analyzed from frozen samples. Although these 4 samples had been frozen for storage, their frequencies of IgG-SCs (mean 0.27% among PBMCs) were comparable to those found on average in fresh PBMCs from the rest of the cohort.

### Aqueous phase I: preparation of cells for droplet compartmentalization

Cell suspensions were centrifuged (300*g*, 5 minutes) and resuspended twice using MACS buffer consisting of PBS pH 7.2, 0.2% bovine serum albumin, and 2 mM EDTA. After each resuspension, cells were filtered through a 40-μm cell strainer to eliminate aggregates. Cells were then spun (300*g*, 5 minutes) and resuspended in DropMap buffer, which consisted of RPMI-1640 (without phenol red) supplemented with 0.1% Pluronic F68, 25 mM HEPES pH 7.4, 5% KnockOut serum replacement (all Thermo Fisher Scientific), and 0.5% human serum albumin (Sigma-Aldrich). Cell number in the suspension was adjusted to achieve a λ (mean number of cells per droplet) of approximately 0.3. For calibration curves, IgG mAbs were diluted in DropMap buffer.

### Aqueous phase II: preparation of beads and bioassay reagents

Paramagnetic nanoparticles (Bio-Adembeads Streptavidin plus 300 nm, Ademtech) were washed with Dulbecco’s PBS with calcium and magnesium (DPBS++, Thermo Fisher Scientific). The nanoparticles were resuspended in DPBS++ containing 1 μM biotin-labeled anti–human κ light chain (Igκ) (CaptureSelect, Thermo Fisher Scientific), and incubated 20 minutes at room temperature. After another wash with DPBS++, nanoparticles were resuspended in 5% Pluronic F127 (Thermo Fisher Scientific) and incubated 20 minutes at room temperature. The nanoparticles were washed again and resuspended in DropMap buffer containing fluorescent reporter proteins at a final concentration of 1.25 mg/mL beads. Reporter proteins were Alexa Fluor 647–labeled F(ab′)_2_ fragment of rabbit anti–human IgG Fc-specific (Jackson ImmunoResearch) used at 75 nM final in-droplet concentration and Alexa Fluor 488–labeled (Thermo Fisher Scientific) αIIbβ3 (purified protein, Enzyme Research) used at 30 nM final in-droplet concentration.

### Droplet production and collection

Droplets were generated using hydrodynamic flow-focusing on a microfluidic chip as described previously (22). The wafer master of an SU-8 photoresist layer (MicroChem) with approximately 40 μm thickness was manufactured using soft lithography (49) and microfluidic chips were fabricated using polydimethylsiloxane (Sylgard; ref. 22). The continuous phase consisted of 2% (wt/wt) 008 Fluorosurfactant (RAN Biotechnologies) in Novec HFE7500 fluorinated oil (3M). Aqueous phases I and II were coflowed and partitioned into droplets. The flow rate of aqueous phases I and II was 70 μL/h, whereas that of oil was 600 μL/h in order to achieve monodisperse droplets of approximately 40 pL volume. Newly generated droplets were directly injected into the DropMap 2D chamber system (22) and mounted on a fluorescence microscope (Ti Eclipse, Nikon). The emulsion was exposed to a magnetic field, forcing the nanoparticles inside each droplet to form an elongated aggregate termed a “beadline.”

### Data acquisition

Images were acquired using a Nikon inverted microscope with a motorized stage (Ti Eclipse). Excitation light was provided by a light-emitting diode (LED) source (SOLA light source, Lumencor Inc.). Fluorescence for the specific channels was recorded using appropriate bandpass filters and camera settings (Orca Flash 4.0, Hamamatsu) at room temperature and ambient oxygen concentration. Images were acquired using a 10× objective (NA 0.45). An array of 10 × 10 images was acquired for each replicate, every 7.5 minutes in all channels over 37.5 minutes (6 measurements total). Duplicates or triplicates were systematically acquired for every sample, with each replicate being the filling of the DropMap 2D chamber with a different droplet population acquired over time on a 10 × 10 image array.

### Image analysis and calculations

Images were analyzed using a custom-made MatLab script (MathWorks) that identifies each droplet and the beadline within each droplet. Fluorescence values associated with the beadline were extracted as well as the mean fluorescence of the entire droplet except the beadline (background fluorescence). A value of fluorescence relocation for each droplet at each time point was calculated, i.e., fluorescence value of the beadline divided by the fluorescence value of the background. Data were exported to Excel (Microsoft) and sorted for droplets showing an increase in relocation of the anti-IgG reporter fluorescence (Alexa Fluor 647) over time and above a threshold of Alexa Fluor 647 relocation greater than 1.5. The sorted droplets were visually controlled for the presence of a single cell within the droplet, for droplet movement between image acquisitions, absence of fluorescent particles other than the beadline (e.g., Alexa Fluor 647–anti-IgG or Alexa Fluor 488–αIIbβ3 protein aggregates, cell debris) and undesired aggregation of fluorescent reporters on the cell surface inside the droplet. IgG secretion rate and dissociation constant (*K_D_*) were estimated as described previously (22).

#### Estimation of IgG concentration within each droplet.

A calibration curve for the estimation of IgG concentration was obtained by preparing droplet populations containing all bioassay reagents except cells that were replaced by a range of concentrations of monoclonal IgG1 (one concentration per droplet population). Images were acquired exactly 3 minutes after the droplets were immobilized in the DropMap chamber for each IgG concentration, and fluorescence relocation calculated. The calibration curve was subsequently used to estimate the IgG concentration and secretion rate (IgG/s) for each time interval, and the mean secretion rate was calculated by averaging the values of all intervals.

#### Estimation of K_D_ for αIIbβ3 within each droplet.

A calibration curve was obtained by defining relocation values for both anti-IgG (Alexa Fluor 647) and αIIbβ3 (Alexa Fluor 488) for a collection of 10 anti-αIIbβ3 mAbs (listed in Supplemental Table 3) with a range of *K_D_* over 1 log as defined using bio-layer interferometry (ForteBio). Droplet populations were generated for different concentrations of each mAb to be analyzed by the DropMap bioassay. A curve was defined by plotting the relocation from the anti-IgG (Alexa Fluor 647) against relocation of αIIbβ3 (Alexa Fluor 488), and the slope of the resulting line was calculated and termed the “DropMap slope.” The calibration curve was defined by the linear relationship between *K_D_* for αIIbβ3 and the DropMap slope of each mAb, and allowed to extract a *K_D_* value for each DropMap slope value calculated from a droplet containing an IgG-SC of unknown affinity for αIIbβ3.

### Affinity determination of mAbs

Bio-layer interferometry measurements were performed using anti–human IgG sensors in an Octet system (ForteBio). Anti-αIIbβ3 mAbs (10 μg/mL) were captured on the sensors for 10 minutes. Equilibrium dissociation constants (*K_D_*) were determined by monitoring over 85 minutes the association between the immobilized antibodies and αIIbβ3 in solution in duplicate for 7 concentrations of antigen (200, 100, 50, 25, 12.5, 6.25, and 3.13 nM). An irrelevant IgG mAb was used as negative control to subtract the background signal. Data analysis was performed using the Octet Analysis software (ForteBio), and *K_D_* values were calculated by steady-state analysis.

### Flow cytometry

Fresh PBMCs were isolated from venous blood samples via standard density gradient centrifugation. For surface staining, cells were washed and resuspended at 2 × 10^6^ in 100 μL PBS with 2% FBS and incubated with Zombie Violet fixable viability dye (BioLegend) and an antibody cocktail for 25 minutes at 4°C in the dark. Following surface staining, cells were fixed/permeabilized for 30 minutes at 4°C in the dark with an eBioscience FoxP3 transcription factor buffer kit and incubated with antibodies recognizing intracellular targets for 30 minutes at 4°C in the dark. Samples were acquired on an LSR Fortessa (BD Biosciences). Data were analyzed with Kaluza software (Beckman Coulter). A list of antibodies used in this panel can be found in Supplemental Table 1 and the detailed gating strategy is depicted in Supplemental Figure 8.

### ELISpot

Anti-αIIbβ3 ELISpot assays were performed as previously described (13). Briefly, KLH (2.5 μg/mL), goat anti–human Ig polyvalent antibody (10 μg/mL; Invitrogen), or purified αIIbβ3 (15 μg/mL; Stago) was coated in PBS and 0.05% CaCl_2_ in multiscreen 96-well filter plates (MSIPS4510, Millipore) with overnight incubation at 4°C.

Then, 1 × 10^6^ splenocytes were serially diluted in culture medium (RPMI-1640 supplemented with 10% HyClone FBS and 1% penicillin/streptomycin) in triplicate before transferring to ELISpot plates and incubated overnight at 37°C with 5% CO_2_. Cells were removed and the ELISpot plate was incubated for 4 hours at 4°C with biotinylated goat anti–human IgG Fc (Invitrogen), followed by incubation for 1 hour at room temperature with horseradish peroxidase–conjugated (HRP-conjugated) avidin (Vector Laboratories). HRP activity was further revealed using 3-amino-9-ethylcarbazole (BD Biosciences) for 8 minutes at room temperature in the dark. Spots were enumerated in each well with an ELISpot reader using AID software v3.5 (AutoImmun Diagnostika).

### Statistics

The R environment v4.0.5 was used for all the analyses (50). Data were neither averaged nor normalized prior to analyses. Response variables were log_2_ converted for better adjustment to linear models. Data were fitted to a linear (quantitative response) or logistic (qualitative response) model that includes the variables of interest, i.e., group (healthy donor or ITP classes) and sample type (SP, BL or BM classes), plus the patient variable nested into the group variable. Group and sample type interaction was added in the model related to Figure 2A because of the post hoc inter-variable contrast comparisons. Otherwise, it was removed from the models when the effect was not significant. Age and sex covariates were not incorporated, as their effects were already represented by the patient variable. Mixed models using the lmer() function of the lme4 package were used in order to consider the effect of patient and patient-group interaction as random. ANOVAs were performed with the Anova() function of the car package. Type 3 sum of squares was applied because of unbalanced designs. Two-by-two effect comparisons (contrast comparisons) were performed with the emmeans() function of the emmeans package. Unequal variance *t* test (Welch’s test) was used in bivariate designs, i.e., when data were analyzed on a single patient (Figure 1) or in the presence of a single value per patient (Figure 3A). In Figure 3, D–F, Pearson’s correlation test was performed after removal of zero values, log_2_ transformation, and residuals checking of the linear regression carried out in both ways. Statistical significance was set to a *P* value of 0.05 or less. In each figure, type I error was controlled by correcting the *P* values according to the Benjamini & Hochberg method [“BH” option in the p.adjust() function of R]. Results are detailed in Supplemental Table 2.

### Study approval

This study was conducted in compliance with the Declaration of Helsinki principles and was approved by the Agence de la Biomédecine and the Institutional Review Boards Comité de Protection des Personnes (CPP) Ile-de-France IX (patients with ITP) and Ile-de-France II (healthy donor spleens). All patients with ITP provided written informed consent before the collection of samples.

## Author contributions

PCH, KE, JB, M Mahevas, and PB designed the experiments. PCH, EC, MB, AS, GC, IA, AV, AP, BI, ORL, CC, GM, AR, and DS carried out the investigations. PCH, MB, PE, GAM, KE, JB, M Mahevas, and PB conducted formal analyses of the data. PCH and PB wrote the original draft of the manuscript. PCH, EC, MB, AS, GC, IA, AV, AP, BI, ORL, CC, GM, DS, AR, M Michel, PE, GAM, KE, JB, M Mahevas, and PB reviewed and edited the manuscript.

## Supplementary Material

Supplemental data

## Figures and Tables

**Table 1:**
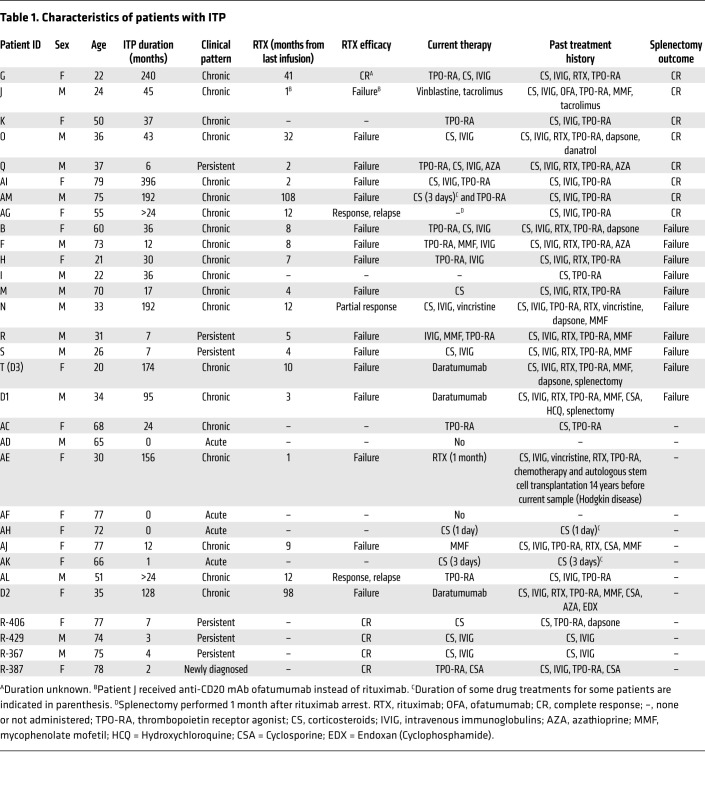
Characteristics of patients with ITP

## Version 1 05/03/2022

In-Press Preview

## Version 2 06/15/2022

Electronic publication
