# Supplementary material for: High-affinity autoreactive plasma cells disseminate through multiple organs in patients with immune thrombocytopenic purpura
Source: J Clin Invest. 2022 Jun 15;132(12):e153580. doi: 10.1172/JCI153580 (PMC9197514; doi:10.1172/JCI153580)
Supplement: Supplemental data [file jci-132-153580-s172.pdf]

Supplementary Materials

Supplementary Figures S1 – S8

Supplementary Tables S1 – S3

Figure S1

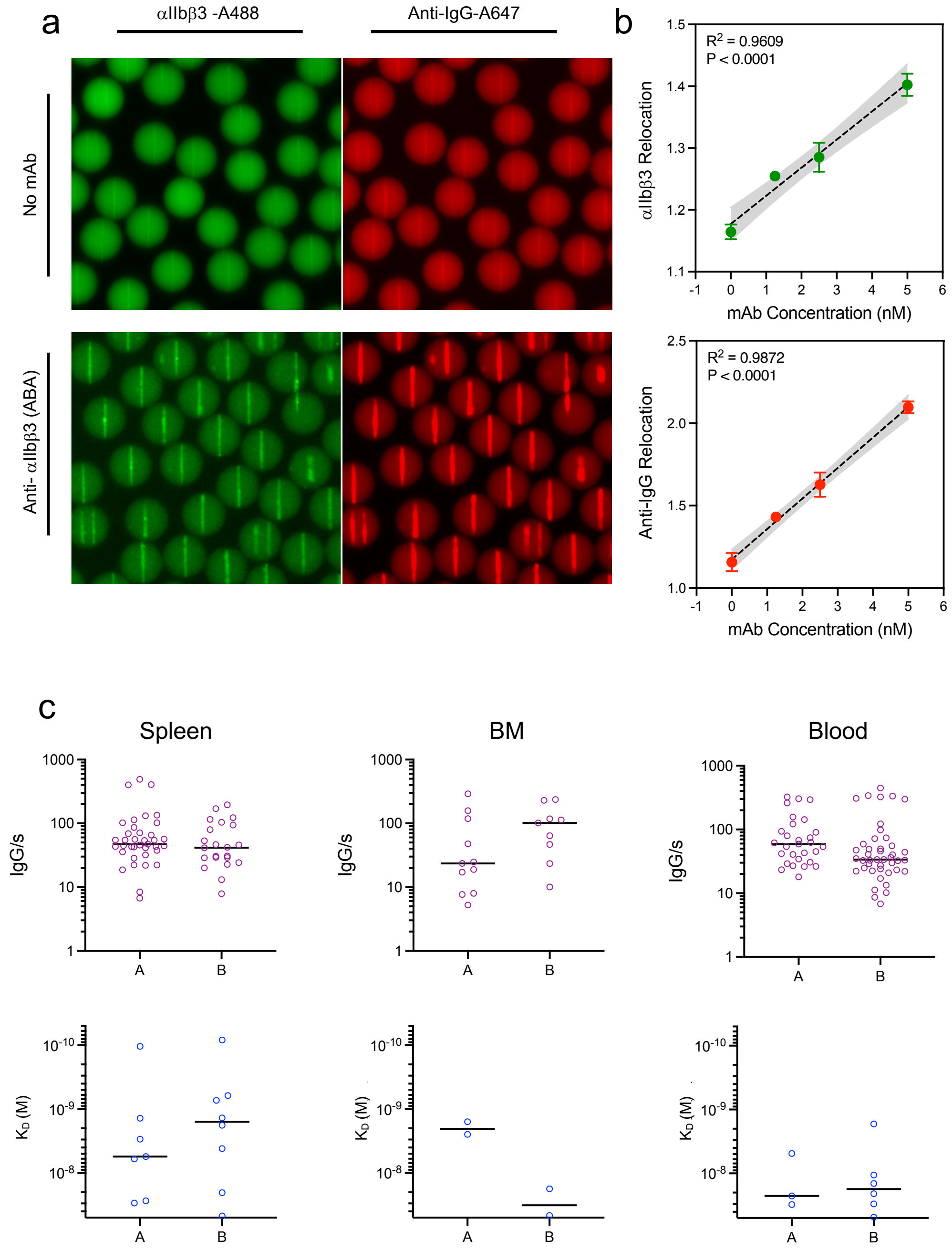

Figure S1. Bioassay calibration and reproducibility. (**a**) Representative images of droplet arrays containing a monoclonal antibody specific for αIIbβ3. (**b**) Quantification of fluorescent relocation in response to antibody concentration for αIIbβ3-Alexa488 (green) and anti-IgG F(ab')2-Alexa647 (red). Fluorescent relocation is obtained by dividing the fluorescent signal from the beadline by the average fluorescent background signal of the drop. Relocation values for a range of monoclonal antibody concentrations were plotted and linear regression was performed. R-squared and P values are indicated. (**c**) Replicates for single-cell measurements. Data for IgG-secretion (top) and affinity for αIIbβ3 (bottom) is shown for two replicates (A and B). All samples described in this work were analyzed in duplicate or triplicate.

Figure S2

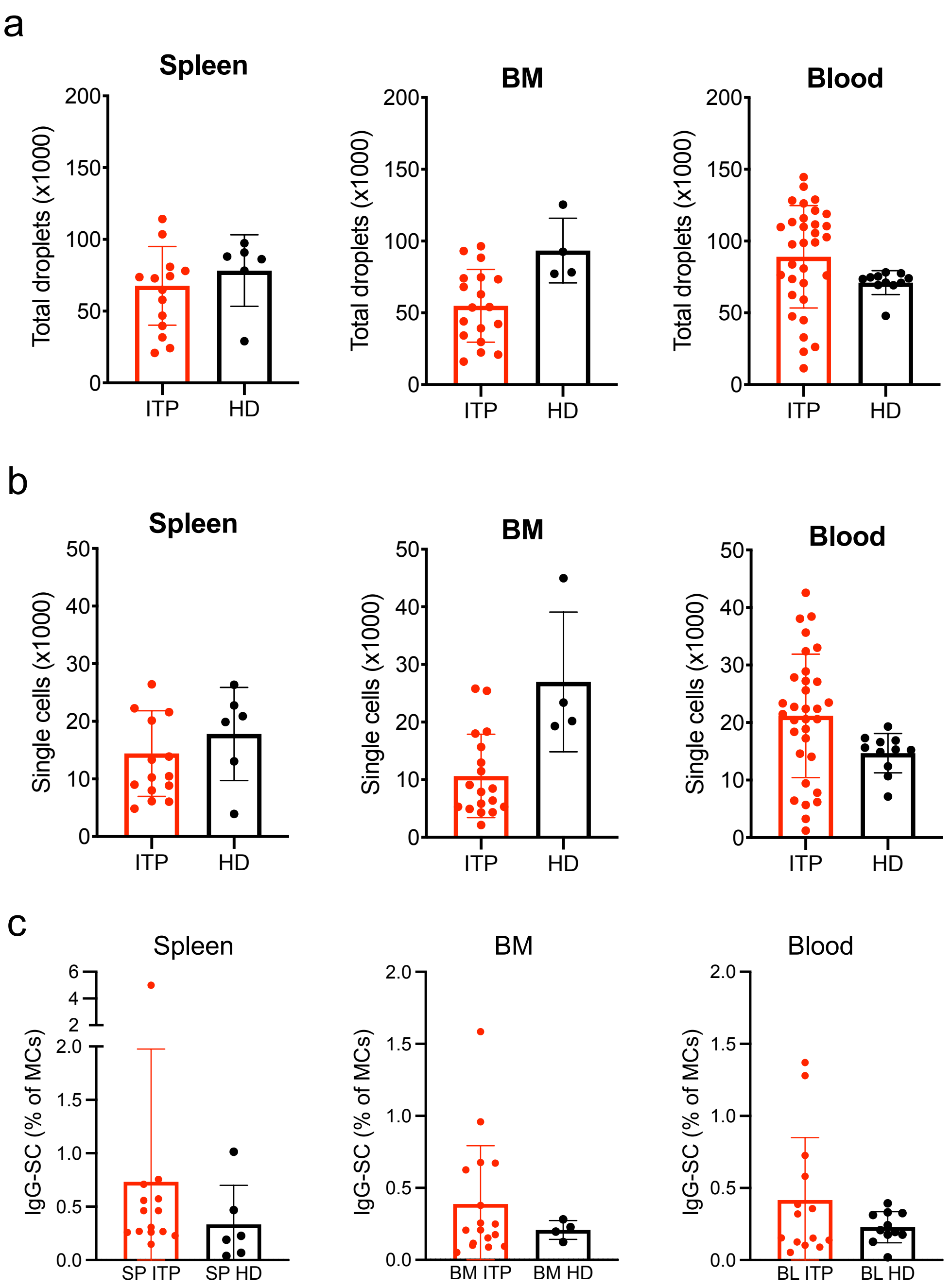

Figure S2. DropMap system throughput and IgG production by single cells. (**a**) Total number of droplets analyzed, (**b**) total number of single cells included in the analysis, and (**c**) number of IgG-SC found in each sample are represented for every sample described in this work. Every data point corresponds to a single sample after pooling of 2-3 replicate acquisitions. Data is shown as mean and SD.

Figure S3

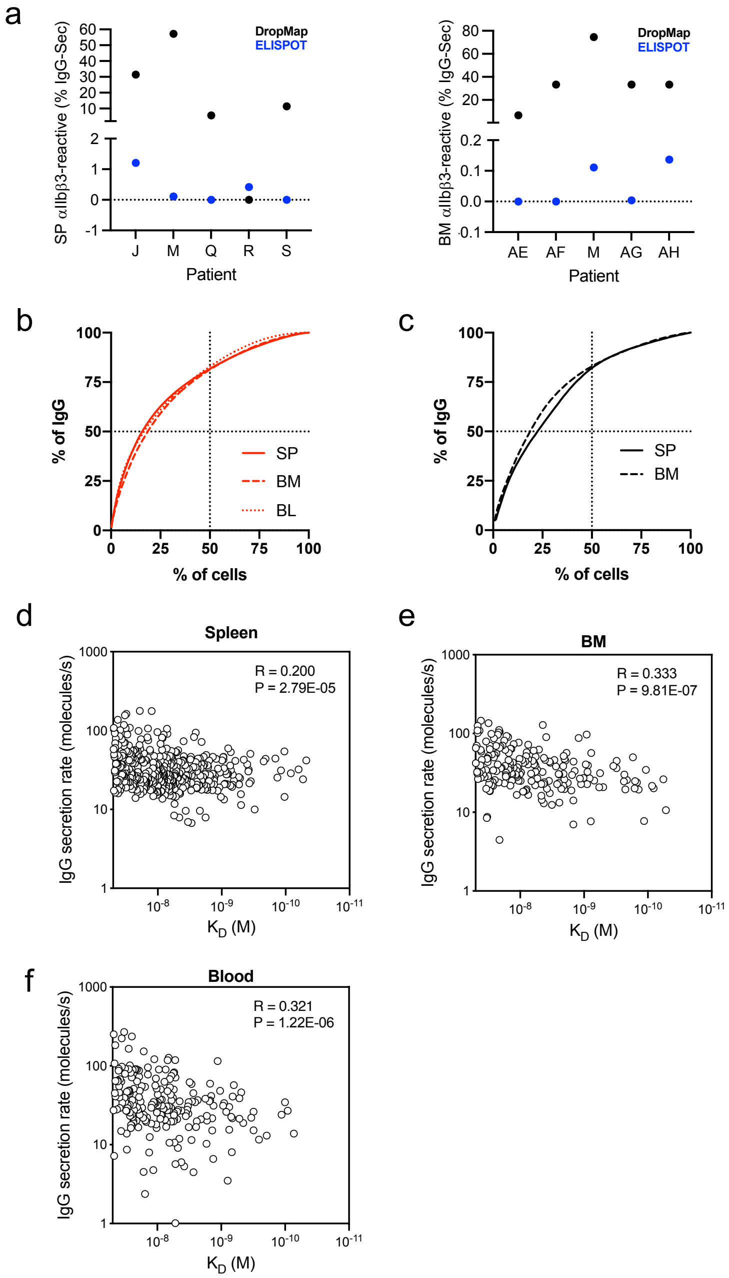

Figure S3. Sensitivity of the bioassay, cumulative IgG secretion and absence of correlation between IgG secretion and affinity for αIIbβ3. (**a**) Comparison of percentage of αIIbβ3-reactive cells among IgG-SC identified by DropMap (black dots) or by ELISPOT (blue dots) in spleen (SP; left) and bone marrow (BM; right). (**b-c**) Percentage of the total mass of secreted IgG relative to the percentage of IgG-SC ordered from highest to lowest IgG producer. Data for all the IgG-SC from all (b) patients with ITP and (c) healthy donors is pooled by organ. (**d-f**) Absence of correlation between IgG secretion rate and K_D for αIIbβ3. Pooled data from all anti-αIIbβ3 IgG-SC identified from all (d) spleen, (e) bone marrow and (f) blood from all patients with ITP is shown. Correlation analysis performed using Pearson's coefficient, R values are indicated.

Figure S4

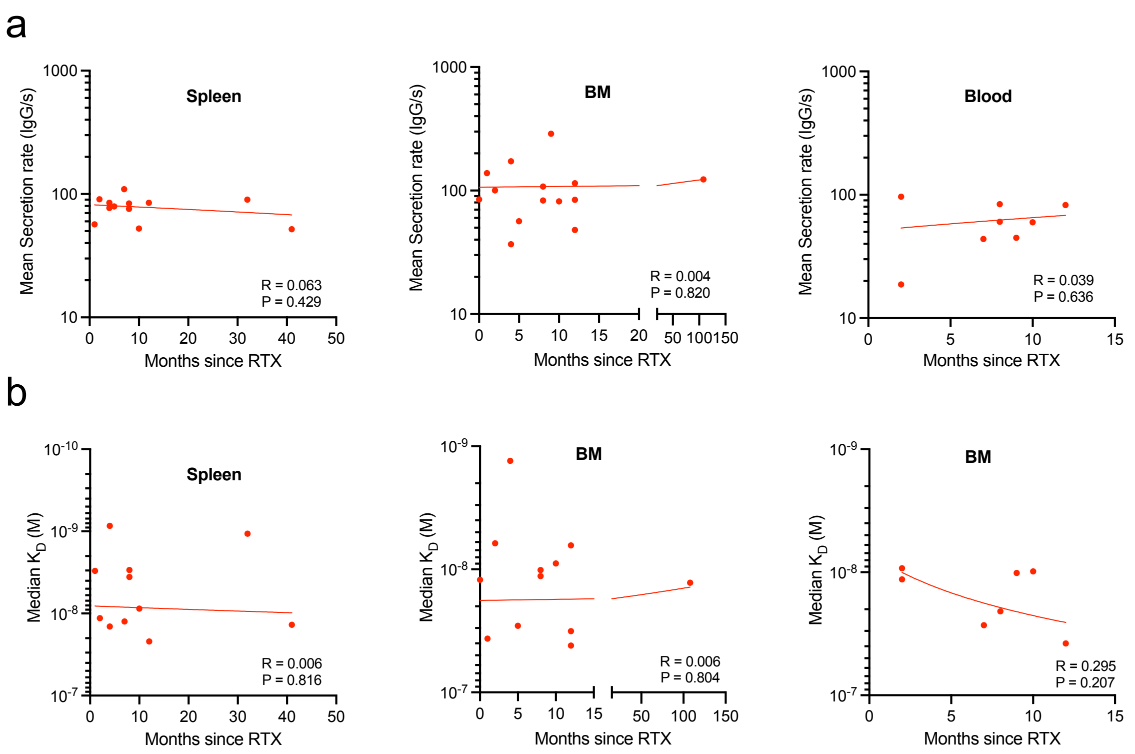

Figure S4. Absence of correlation between last rituximab infusion and secretion rate or anti-αIIbβ3 affinity. Scatterplots compare the mean secretion rate (a) or the Median K_D (b) with the time since the last Rituximab infusion. Cells were obtained from spleen (left), bone marrow (center) and blood (right) of ITP patients. Pearson analysis was performed, R and P values are indicated.

Figure S5

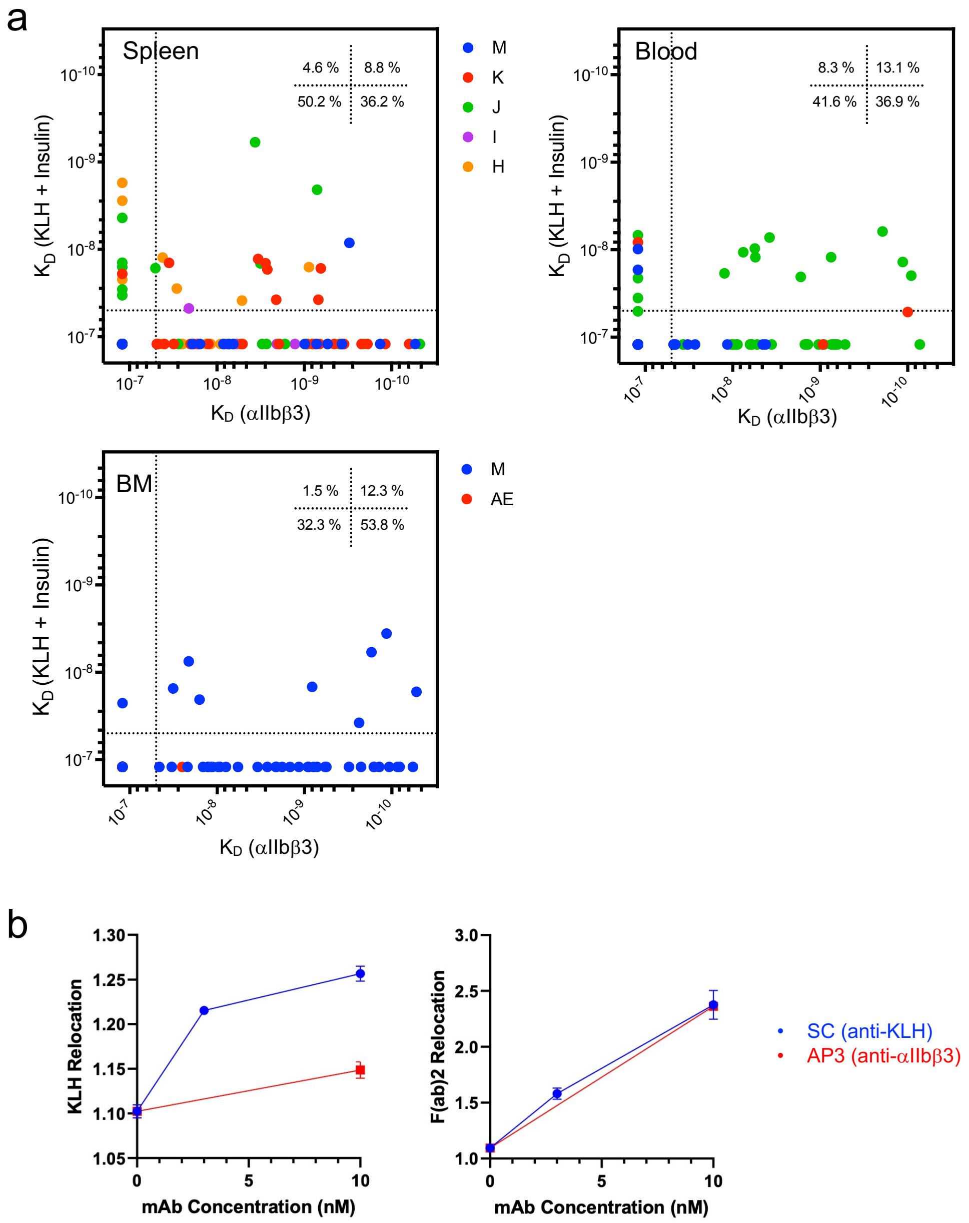

Figure S5. Polyreactivity measurements. (**a**) Affinity values of single IgG-SC against αIIbβ3 and (KLH+insulin) from a multiplexed bioassay containing fluorescently labeled αIIbβ3-Alexa488, KLH-Alexa405 and Insulin-Alexa405. KLH and human insulin are labeled with the same fluorochrome to obtain a single affinity measurement [K_D (KLH+insulin)] for both these irrelevant antigens as a measure of polyreactivity. Distribution per quadrant is indicated with color codes per patient. (**b**) Positive and negative control for KLH binding using an anti-KLH IgG mAb (clone SC) and an anti-αIIbβ3 IgG mAb (clone AP3) using the polyreactivity bioassay described in (b). Relocation of KLH (left) and anti-IgG are shown at different concentrations of the mAbs.

Figure S6

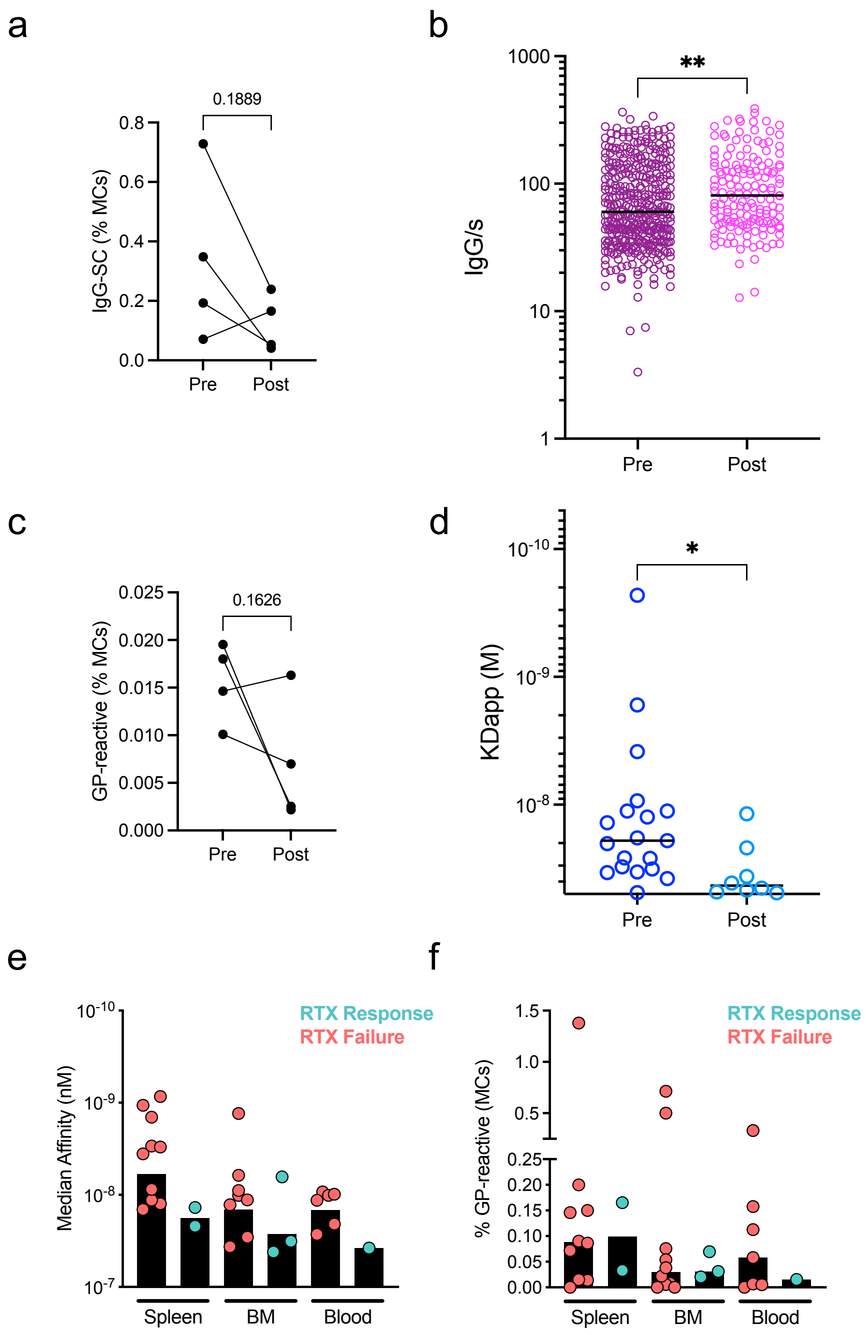

Figure S6. Effects of Rituximab therapy on the anti-αIIbβ3 ASC population. (**a-d**) Kinetic follow-up of four RTX-responding patients before (pre) and after (post) rituximab therapy for (**a**) frequency of IgG-SC among PBMCs, (**b**) IgG secretion rate, (**c**) frequency of αIIbβ3-reactive IgG-SC among PBMCs, (**d**) affinity for αIIbβ3 of single IgG-SC. Single-cell secretion rates and affinity values, and medians, are plotted in (b) and (d), respectively. (**e**,**f**) Patients from the main cohort (all patients except the four RTX-responding patients) were grouped according to Rituximab responsiveness: (**e**) Median affinity and (**f**) median frequency of autoreactive cells at the time of sampling. $^*P < 0.05$; $^{**}P < 0.01$.

Figure S7

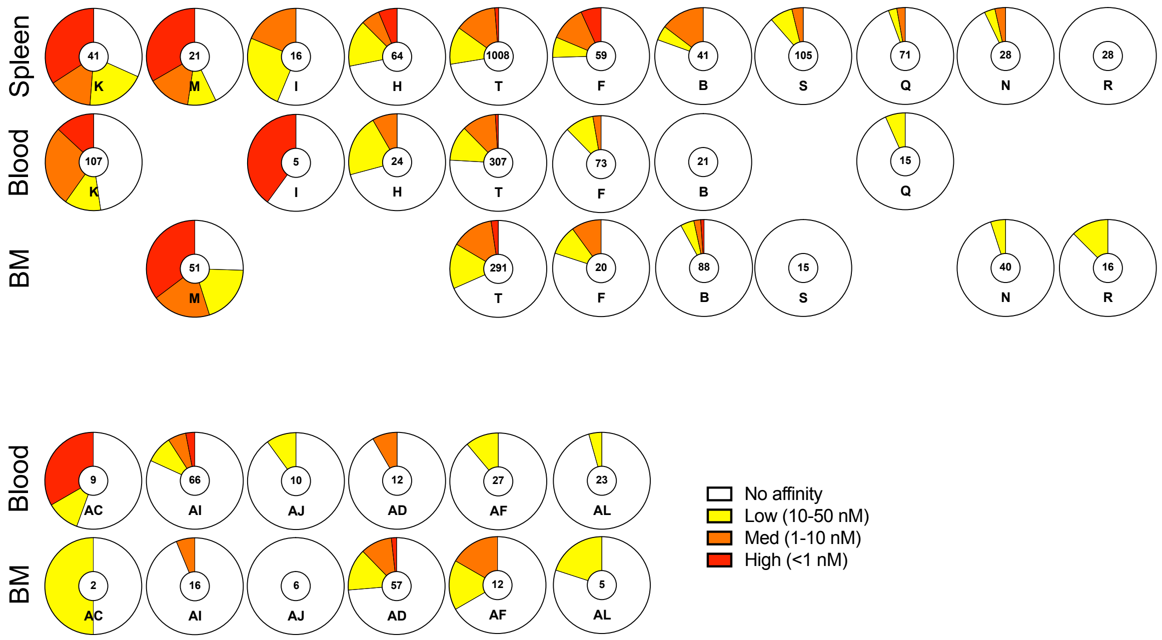

Figure S7. Affinity distribution among paired organs. Distribution of IgG-SC into low (yellow), medium (orange) and high (red) affinity binders to αIIbβ3 or non-binders (white), with total IgG-SC numbers indicated, for all patients with ITP with paired samples in this study. Samples are represented ordered by the proportion of αIIbβ3-reactive cells present in the spleen (top) or blood (bottom).

Figure S8

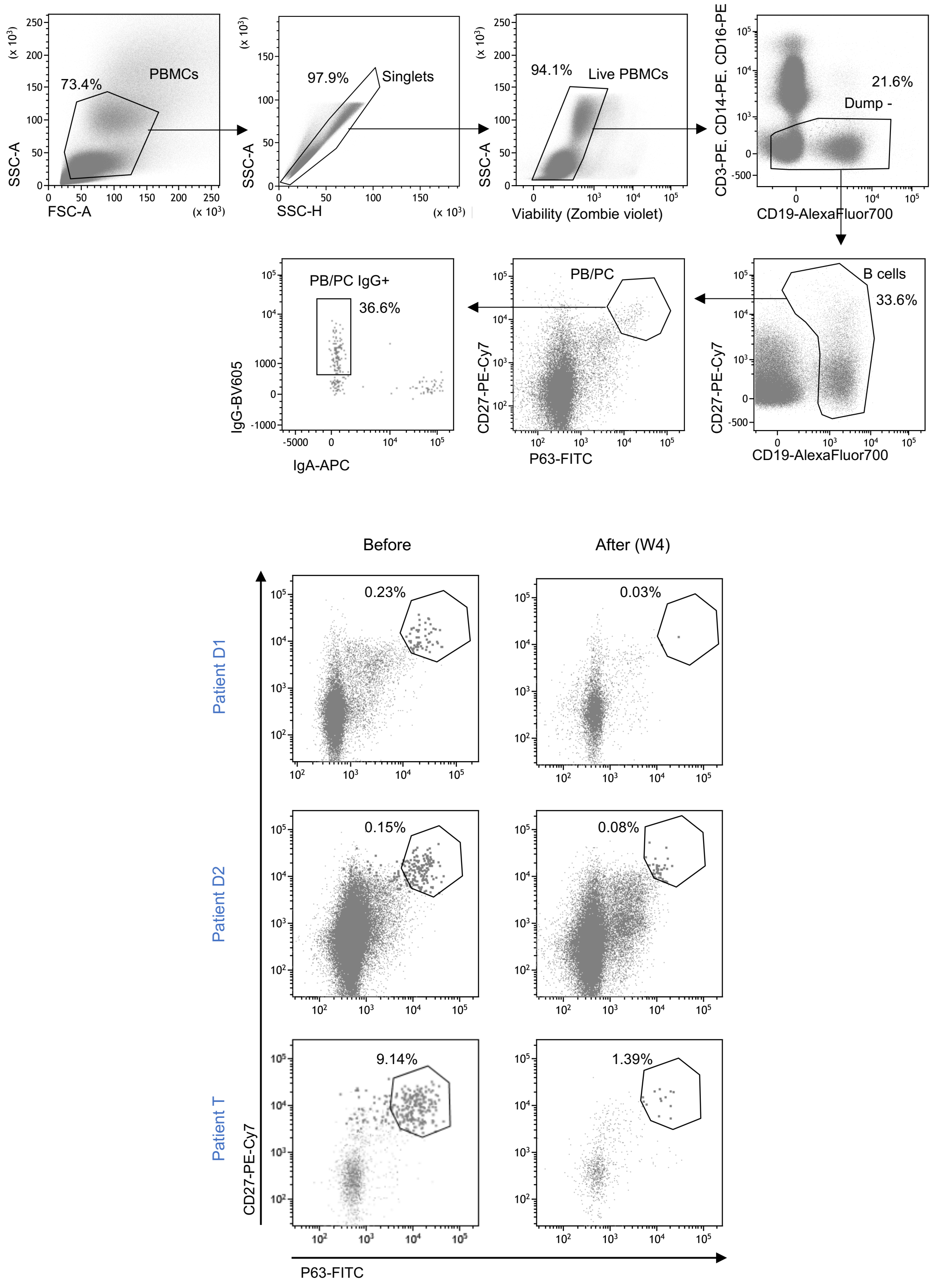

Figure S8. Identification of circulating plasma cells following daratumumab therapy. (Top) Gating strategy is shown for IgG$^+$ plasma cell for a representative PBMC sample after Ficoll gradient and B cell enrichment by negative selection of CD3$^+$ cells. Plasma cells are identified as live CD3$^-$CD14$^-$CD16$^-$ CD19$^{+/-}$CD27$^+$p63$^+$ cells and IgG$^+$ cells. Percentages of cells within each gate are indicated. All antibodies used for this strategy are included in Supplementary Table 1. (Bottom) Follow-up of circulating IgG$^+$ plasma cells (within the gate) before daratumumab therapy and at week 4 after the first infusion of daratumumab in patients D1, D2 (same data and dot plots as in Fig.4a) and T. Percentages of cells within each gate are indicated.

Table S1. Reagents and software used for flow cytometry.

Reagent	Source	Identifier	Fluorochrome
**Antibody for surface staining**			
CD3	BD Bioscience	UCHT1 ; 555333	PE
CD14	BD Bioscience	M5E2 ; 564054	PE
CD16	BD Bioscience	3G8 ; 560995	PE
CD38	BD Bioscience	HIT2 ; 551400	PerCP Cy5.5
CD27	BD Bioscience	M-T271 ; 560609	PE-Cy7
CD19	BD Bioscience	HIB19 ; 557921	AF700
IgD	BD Bioscience	IA6-2 ; 562540	PE-CF594
**Antibody for intracellular staining**			
VS38c	Dako	VS38c ; F7149	FITC
IgG	BD Bioscience	G18-145 ; 563246	BV605
IgA	Miltinyi Biotec	IS11-8E10 ; 130-113-472	APC
IgM	BD Bioscience	G20-127 ; 563113	BV510
**Chemical**			
eBioscience™ Foxp3 / Transcription Factor Staining Buffer Set	ThermoFisher	00-5523-00	
Zombie Violet™ Fixable Viability Kit	Biolegend	423114	
Softwares			
Kaluza v2.1	Beckman Coulter		
GraphPad Prism v8	GraphPad		

Table S2. Statistical analyses[a].

Figure	Comparison	Alternative hypothesis	Sample size	Transformation	Test	p-value	Adjusted p-value
1C	SP and BM	mean difference	n = 59, n = 20	log	Welch test	0.76	0.97
	SP and BL	mean difference	n = 59, n = 73	log	Welch test	0.64	0.97
	BM and BL	mean difference	n = 20, n = 73	log	Welch test	0.97	0.97
1D	SP and BM	mean difference	n = 15, n = 4	log	Welch test	0.33	0.50
	SP and BL	mean difference	n = 15, n = 9	log	Welch test	0.01	0.04
	BM and BL	mean difference	n = 4, n = 9	log	Welch test	0.50	0.50
2A	ITP and HD in SP	effect difference	n = 1592, n = 399 | df = 44.9	log	Contrast test in linear model	6e-6	1e-5
	ITP and HD in BM	effect difference	n = 706, n = 206 | df = 38.8	log	Contrast test in linear model	0.63	0.70
	ITP and HD in BL	effect difference	n = 712, n = 376 | df = 43.7	log	Contrast test in linear model	1e-7	4e-7
	SP and BM in ITP	effect difference	n = 1592, n =706 | df = 3787.7	log	Contrast test in linear model	5e-17	4e-16
	SP and BL in ITP	effect difference	n = 1592, n =712 | df = 3882.1	log	Contrast test in linear model	0.27	0.34
	BM and BL in ITP	effect difference	n = 706, n = 712 | df = 3785.0	log	Contrast test in linear model	6e-16	3e-15
	SP and BM in HD	effect difference	n = 399, n = 206 | df = 40.2	log	Contrast test in linear model	0.06	0.10
	SP and BL in HD	effect difference	n = 399, n = 376 | df = 43.3	log	Contrast test in linear model	0.79	0.79
	BM and BL in HD	effect difference	n = 206, n = 376 | df = 38.1	log	Contrast test in linear model	0.07	0.10
2B	ITP and HD in SP	effect difference	n = 1592, n = 399 | df = 40.4	log	Contrast test in linear model	0.005	0.005
	ITP and HD in BM	effect difference	n = 706, n = 206 | df = 34.2	log	Contrast test in linear model	0.005	0.005
	ITP and HD in BL	effect difference	n = 712, n = 376 | df = 38.6	log	Contrast test in linear model	0.005	0.005
2C	ITP and HD in SP	effect difference	n = 1592, n = 399 | df = Inf	log	Contrast test in linear model	0.006	0.006
	ITP and HD in BM	effect difference	n = 706, n = 206 | df = Inf	log	Contrast test in linear model	0.006	0.006
	ITP and HD in BL	effect difference	n = 712, n = 376 | df = Inf	log	Contrast test in linear model	0.006	0.006
3A	SP and BM	mean difference	n = 14, n = 17	none (0 values)	Welch test	0.4694	0.7041
	SP and BL	mean difference	n = 14, n = 14	none (0 values)	Welch test	0.2987	0.7041
	BM and BL	mean difference	n = 17, n = 14	none (0 values)	Welch test	0.7416	0.7416
3D-F	SP and BL	Correlation different from zero	n = 6	log (0 values removed)	Pearson correlation test	0.007	0.02
	SP and BM	Correlation different from zero	n = 5	log (0 values removed)	Pearson correlation test	0.02	0.03
	BM and BL	Correlation different from zero	n = 7	log (0 values removed)	Pearson correlation test	0.58	0.58
S1-B	mAb and Relocation (GPIIbIIIa)	Slope different from zero	n = 4	none	Simple linear regression	0.000019	-
	mAb and Relocation (IgG)	Slope different from zero	n = 4	none	Simple linear regression	6.6E-07	-
S3-D-F	Kd and IgG-Sec (SP)	Correlation different from zero	n = 431	none	Pearson correlation test	2.79E-05	2.79E-05
	Kd and IgG-Sec (BM)	Correlation different from zero	n = 206	none	Pearson correlation test	9.81E-07	9.81E-07
	Kd and IgG-Sec (BL)	Correlation different from zero	n = 219	none	Pearson correlation test	1.22E-06	1.22E-06
S4-A	Months since RTX and IgG-Sec (SP)	Correlation different from zero	n = 11	none	Pearson correlation test	0.429	-
	Months since RTX and IgG-Sec (BM)	Correlation different from zero	n = 12	none	Pearson correlation test	0.82	-
	Months since RTX and IgG-Sec (BL)	Correlation different from zero	n = 7	none	Pearson correlation test	0.636	-
S4-B	Months since RTX and Kd (SP)	Correlation different from zero	n = 11	none	Pearson correlation test	0.816	-
	Months since RTX and Kd (BM)	Correlation different from zero	n = 12	none	Pearson correlation test	0.804	-

	Months since RTX and Kd (BL)	Correlation different from zero	n = 7	none	Pearson correlation test	0.207	-
S6-A	Pre and Post (Mean IgG-Sec)	mean difference	n = 8	none	Paired T test	0.1889	-
S6-B	Pre and Post (IgG-Sec)	mean difference	n = 550	none	Welch test	0.009	-
S6-C	Pre and Post (Mean GP-Reactive)	mean difference	n = 8	none	Paired T test	0.1626	-
S6-D	Pre and Post (Kd)	mean difference	n = 27	none	Welch test	0.011	-

[a] n: sample size for each class tested, respectively; df: degree of freedom. Adjusted p values from the same panel according to Benjamini & Hochberg

Table S3. Antibodies used for assay calibration

Clone/Identifier	Affinity for αIIbβ3 (K_D - M)	Source	Description
**7E3**	2.6E-10	Absolute antibody	Human Anti-CD41 [Clone 7E3 (Abciximab)]
**H3**	6.0E-08	Institut Necker Enfants Malades, Paris, France (Dr Matthieu Mahévas)	Cloned and expressed in house as a fully human IgG1
**VI-PL2**	1.1E-09	Sony Europe BV	Purified anti-human CD61 (Clone VI-PL2)
**PL2-49**	1.4E-09	BioCytex, France (Dr Maxime Moulard)	anti-CD41
**AP3**	1.8E-09	Institut Pasteur, Paris, France (Dr Pierre Bruhns)	Cloned and expressed in house as a human IgG1 from the published sequence; *Newman PJ, Allen RW, Kahn RA & Kunicki TJ. Blood (1985)*
**HIP2**	8.0E-10	BD Biosciences	CD41b Hu - clone HIP2
**PM3G5**	1.5E-09	BioCytex, France (Dr Maxime Moulard)	anti-CD61 – clone PM3G5
**LYP18**	7.4E-10	Faculté de Médecine René Laënnec, Lyon, France (Dr Habib Boukerche)	mAb purified from clone LYP18 (P18)
**A2A9/6**	4.6E-09	Sony Europe BV	Purified anti-human CD41/CD61
**PM6/13**	1.0E-09	Southern Biotech	Mouse anti-human CD61-UNLB
